# New Potent Inhibitor of Transforming Growth Factor-Beta (TGFβ) Signaling that is Efficacious against Microsatellite Stable Colorectal Cancer Metastasis in Combination with Immune Checkpoint Therapy in Mice

**DOI:** 10.1021/acsptsci.4c00374

**Published:** 2024-10-01

**Authors:** Daniele V. F. Tauriello, Elena Sancho, Daniel Byrom, Carolina Sanchez-Zarzalejo, Maria Salvany, Ana Henriques, Sergio Palomo-Ponce, Marta Sevillano, Xavier Hernando-Momblona, Joan A. Matarin, Israel Ramos, Irene Ruano, Neus Prats, Eduard Batlle, Antoni Riera

**Affiliations:** † Institute for Research in Biomedicine (IRB Barcelona), the Barcelona Institute of Science and Technology (BIST), Baldiri i Reixac 10, Barcelona 08028, Spain; ‡ Centro de Investigación Biomédica en Red de Cáncer (CIBERONC), Barcelona 08028, Spain; § Department of Medical Oncology, Erasmus MC Cancer Institute, University Medical Center Rotterdam, Dr. Molewaterplein 40, 3015 GD Rotterdam, The Netherlands; ∥ Department Química Inorgànica i Orgànica, Universitat de Barcelona, Martí i Franquès 1, Barcelona 08028, Spain; ⊥ Institució Catalana de Recerca i Estudis Avançats (ICREA), Barcelona 08010, Spain; □ Universitat de Barcelona, Barcelona 08028, Spain

**Keywords:** colorectal cancer, TGFβ, metastasis, ALK5 inhibitor, immune checkpoint, anti-PD1

## Abstract

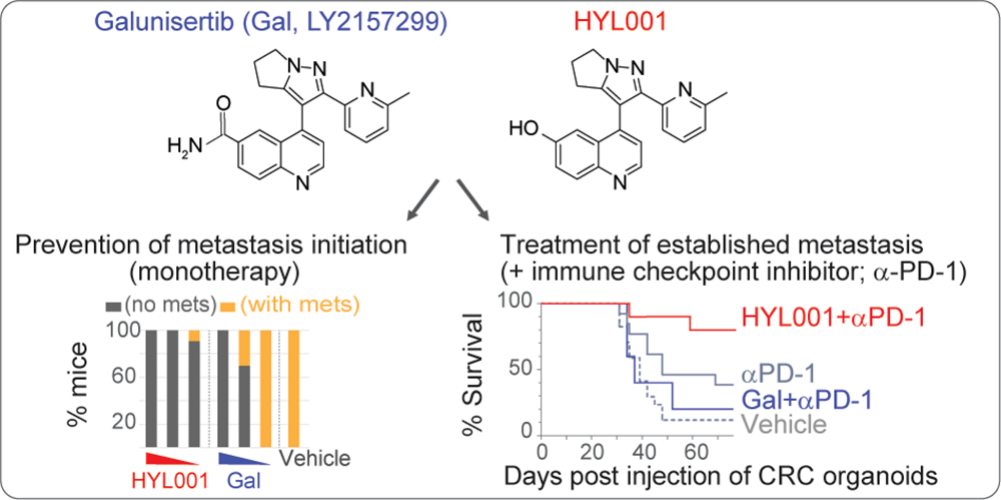

Blockade of the TGFβ signaling pathway has emerged from preclinical studies as a potential treatment to enhance the efficacy of immune checkpoint inhibition in advanced colorectal cancer (CRC) and several other types of cancer. However, clinical translation of first-generation inhibitors has shown little success. Here, we report the synthesis and characterization of HYL001, a potent inhibitor of TGFβ receptor 1 (ALK5), that is approximately 9 times more efficacious than the structurally related compound galunisertib, while maintaining a favorable safety profile. HYL001 in combination with immune checkpoint blockade (anti-PD1) eradicates liver metastases generated in mice by microsatellite stable, aggressive colorectal cancer tumors at doses where galunisertib is ineffective.

Cancer takes almost 10 million lives each year.^1^ Advanced disease with tumors spreading to distant organs (*i.e.*, metastasis) is associated with most cancer deaths. For example, despite considerable progress, metastatic colorectal cancer (mCRC) still has a low 5 year survival rate (<20%) and is thus in urgent need for better treatment options. Among promising recent developments in cancer treatment are immunotherapies, notably immune checkpoint inhibitors (ICIs).^2,3^ However, many patients remain unresponsive to immune therapies. In the case of mCRC, responses seem limited to a small fraction of patients who have tumors that are classified as mismatch-repair deficient or microsatellite-instable (dMMR/MSI). These typically have a high tumor mutational burden that can elicit specific immune responses. Boosting the efficacy of immunotherapy in larger groups of patients would represent a clinical advancement with a tremendous impact.

In the past decades, fundamental evidence has mounted for TGFβ signaling as an immunosuppressive pathway with key relevance in cancer.^4^ Indeed, tumors displaying a TGFβ-activated stroma represent a clinical entity associated with poor prognosis and immune evasion.^5−9^ In 2018, we and others showed that increased activity of TGFβ in the tumor microenvironment (TME) is associated with a lack of response to anti-PD-1/anti-PD-L1 in preclinical CRC models and in human metastatic urothelial cancer.^5,10^ We demonstrated that TGFβ blockade synergizes with immune checkpoint blockade to cure mice with multiple colorectal cancer liver metastases.^5^ Interestingly, our murine mCRC model is mismatch-repair proficient (pMMR), microsatellite stable (MSS), and represents the majority of tumors that, especially when KRAS mutant, have not seen many new efficacious treatment options recently. Additional corroborating studies have been reported since, and clinical trials have begun testing this or similar TGFβ inhibition-based immuno-oncology strategies in a range of cancer types.^6,7^ Furthermore, TGFβ blockade could also be useful to treat additional diseases where this cytokine plays a role, such as fibrosis in vital organs like the lung, liver, kidney, and skin.^11,12^

Despite all this and the development of various agents to inhibit TGFβ signaling, there are no clinically approved drugs to inhibit this pathway.^6,13^ The development of TGFβ inhibitors has been hampered in part by their associations with on-target cardiac toxicity in rats,^14^ which may likely translate to humans and therefore pose limits for drug dosing. Within a class of quinoline-substituted dihydropyrrolopyrazoles, galunisertib (LY2157299) is a small molecule inhibitor that targets the TGFβ signaling pathway by competitively binding the ATP pocket of the TGFβ receptor type I (TGFBR1; ALK5).^15,16^ Given its relatively low toxicity profile, galunisertib has been extensively tested in clinical oncology studies to inhibit TGFβ signaling.^6,17^ This favorable safety profile appears related to a relatively low potency. Accordingly, the aforementioned efficacy against murine CRC liver metastasis required elevated doses of this drug.^5^

An efficacious murine dose for galunisertib was established in our model at 720–800 mg/kg twice daily (BID), which is markedly higher than previously reported.^15^ Although our dosing came with practical challenges, it caused only minor adverse effects in mice. Yet, such elevated doses would very likely not be tolerable in humans. We therefore sought to design a new TGFβ inhibitor with a better potency–safety profile. Furthermore, we aimed for the improved molecule to have a “handle” to which we could attach a variety of groups, enabling further tuning of pharmacochemical properties. Moreover, this could enable a caged prodrug with a spatiotemporally controlled release. In parallel, other second-generation ALK5 kinase inhibitors have been developed that appear to maintain acceptable toxicity despite a higher potency. One of these, vactosertib (TEW-7197),^18^ is currently undergoing clinical investigation for treatment of various solid tumors.^6,13^

## Results

### Chemical Synthesis of HYL001

**Figure 1:**
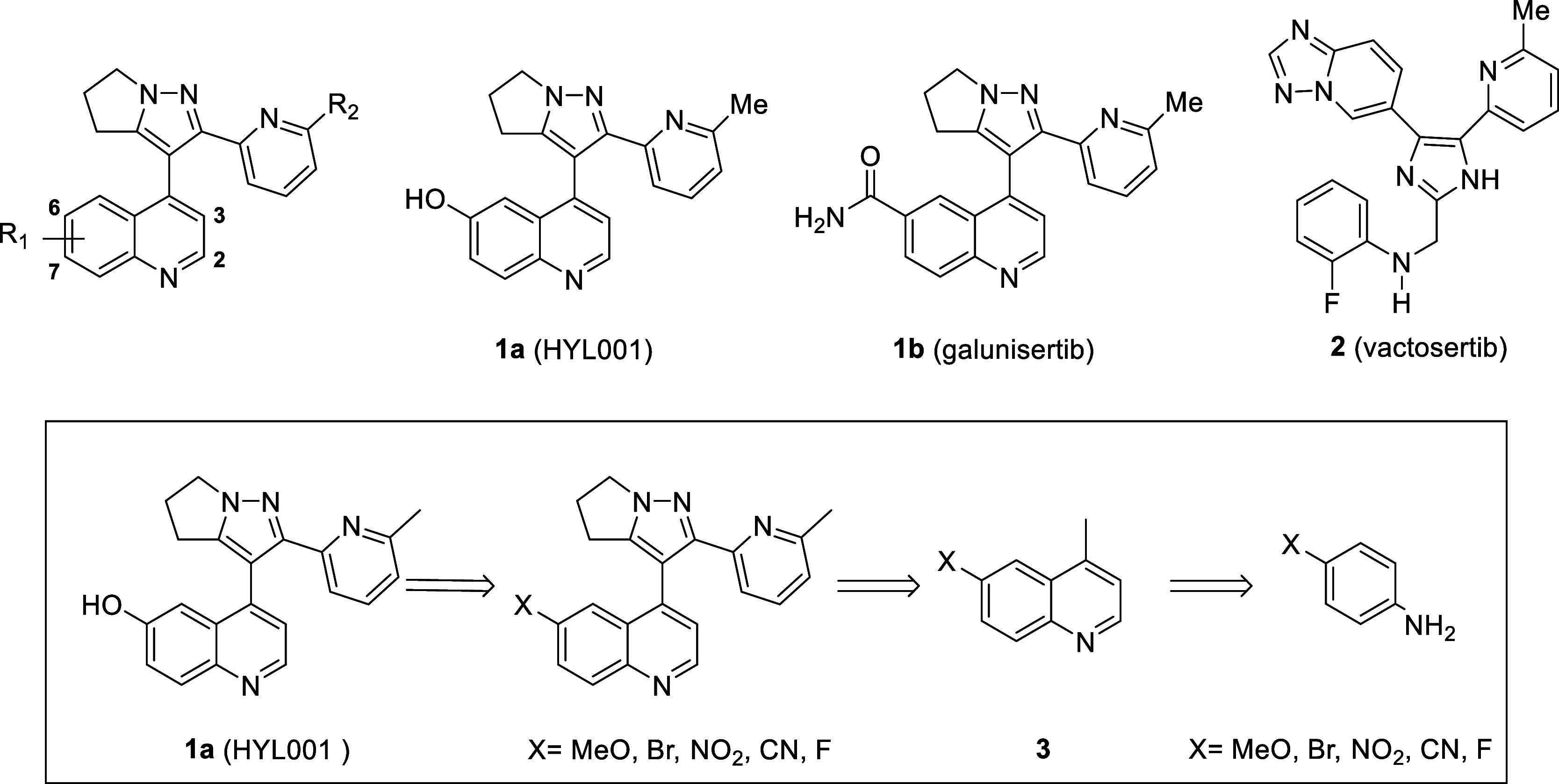
Chemical structures of ALK5 inhibitors. Above: HYL001 (**1a**), galunisertib (**1b**), and vactosertib (**2**). Below: retrosynthetic analysis of HYL001.

In 2008, Li *et al.* published a structure-activity relationship study on the quinoline domain of the 1 series of ALK5 inhibitors.^16^ From this study, it was apparent that the substitution at position 2 had a negative impact on the IC_50_ value when changed for anything but a hydrogen atom. On the other hand, derivatization at the 7-position produced some potent inhibitors. Interestingly, a hydroxyl group at 7-position (7-OH, R_2_=H) afforded a clearly less potent compound (IC_50_, 160 nM) than galunisertib (51 nM).^17^ However, modification at the 6-position appeared to be a promising starting point for us, as the 6-bromo compound (6-Br, R_2_=CH_3_) gave a lower IC_50_ (89 nM). Therefore, we synthesized this previously unreported compound with a hydroxyl group at the 6-position **1a**, which we called HYL001 (Figure 1).

The synthesis of HYL001 was based on the same approach as galunisertib. The target compound should be prepared from compounds bearing a functional group X on the 6-position that could be transformed into the desired phenol. These compounds would be prepared from the corresponding 6-substituted-4-methylquinolines (**3**), easily accessible by a Doebner–Miller reaction of methyl vinyl ketone and the corresponding aniline (Figure 1).

**Scheme 1:**
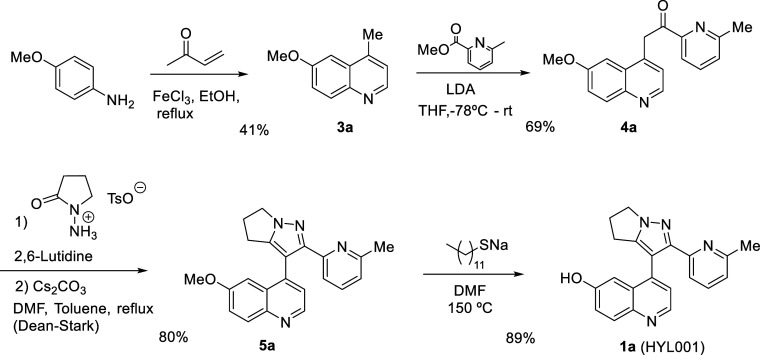
Synthesis of HYL001 from 4-Methoxyaniline

Although the use of nitro, fluoro, and cyanoquinolines was explored, none of these approaches gave good yields of the desired product. Gratifyingly, the use of a methoxy group as a protected phenol was successful, allowing us the preparation of HYL001 (Scheme 1). However, 6-methoxy-4-methylquinoline (**3a**) was an inconvenient starting material. The yield of the Doebner–Miller reaction was low (41%), the product was difficult to purify due to a large amount of ferric salts (chromatography was needed), and LDA was necessary for the alkylation with methyl 6-methylpicolinate to give compound **4a**. Nevertheless, the construction of the dihydropyrrolopyrazole ring to give **5a** as well as the deprotection of the methyl ether went as expected, affording **1a** (HYL001) in good overall yield (Scheme 1).

**Scheme 2:**
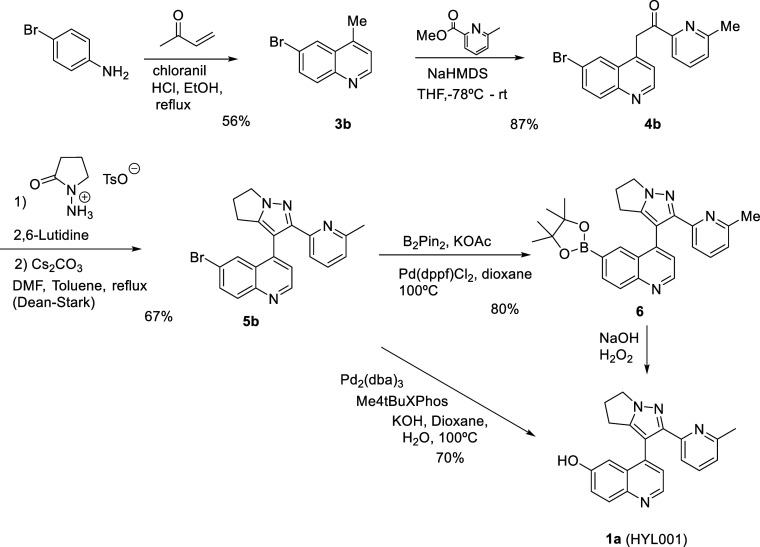
Synthesis of HYL001 from 4-Bromoaniline

The synthesis of HYL001 was next approached starting from 4-bromoaniline (Scheme 2). Its preparation by the Doebner–Miller reaction was more convenient since it could be done without the need of FeCl_3_, and the purification was greatly improved. The subsequent alkylation was performed using HMDS as a base, affording **3b** in excellent yield. Preparation of **4b** took place uneventfully. However, the conversion of bromo derivative **4b** into phenol was more difficult than expected. We eventually found that **5b** could be converted into boronate **6** by Miyaura borylation. Oxidation and hydrolysis of the boronate gave the desired HYL001 in good yield. However, the procedure required a chromatographic purification of **5b** that prevented large-scale synthesis. The procedure could be further improved by the direct conversion of **5b** into the final compound using tris(dibenzylideneacetone)dipalladium(0) and tetramethyl di-tBuXPhos as the ligand. In this way, a convenient and scalable procedure allowed the preparation of multigram quantities of HYL001 without any chromatographic purification.

Both **1a** and **1b** are poorly soluble in water. Their solubility can be improved using a solution of PBS/2-hydroxypropy-β-cyclodextrin (1 g/mL). The solubilities in this solution are 0.7 mg/mL (**1b**) and 0.23 mg/mL (**1a**). In most *in vivo* experiments, a homogeneous suspension in the following vehicle was used: sodium carboxymethylcellulose (1%), sodium dodecyl sulfate (0.4%), polyvinylpyrrolidone (0.085%), and antifoam-A (0.05%). The solubilities in this vehicle are approximately 0.15 mg/mL (**1b**) and 0.09 mg/mL (**1a**). As the solubility of **1a** and **1b** in water formulations is low, a more polar prodrug formulation might benefit administration at least in the preclinical setting. Therefore, as a proof-of-concept, we leveraged the hydroxyl handle to design HYL002. We added a carbonyl-4-piperidinopiperidine group, in analogy to the ester that solubilizes SN-38 into irinotecan, and that can be cleaved *via* hydrolysis inside cells^19^ (Figure S1). HYL001 reacted with commercially available 4-piperidinopiperidine-1-carbonyl chloride in chloroform using triethylamine and catalytic amounts of DMAP (Scheme S1). We then transformed the resulting compound HYL002 into the corresponding HCl salt to aid the *in vivo* drug formulation. The free base was dissolved in dioxane under N_2_, where it was treated dropwise with 10 equiv of 4 M HCl in dioxane at room temperature with vigorous stirring. The reaction mixture was then taken to dryness *in vacuo*, giving the HCl salt of HYL002.

### Selectivity Profile of HYL001

The *in vitro* binding affinity of HYL001 toward selected members of the TGFβ receptor family was assessed using a competitive binding assay. HYL001 is a potent ALK4 and ALK5 inhibitor with a *K*_d_ of 13 and 22.5 nM, respectively (Table S1). In comparison, the reported values for galunisertib are around 78 and 172 nM, respectively.^15^ However, in the same assay as performed for HYL001, we found a *K*_d_ of 52 nM for galunisertib against ALK5. We also included vactosertib, which gave an ALK5 *K*_d_ value of around 4 nM (Table S1).

**Figure 2:**
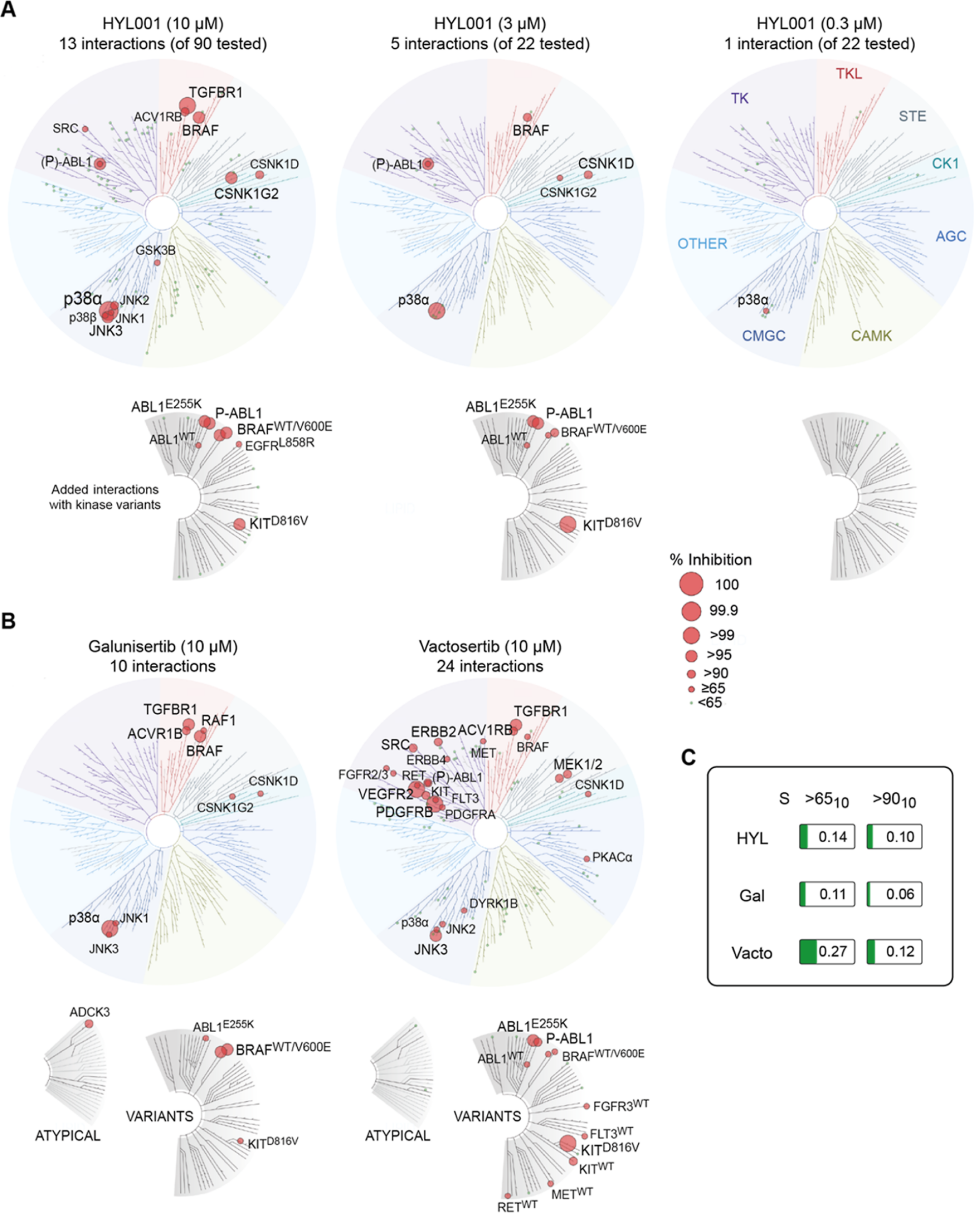
Selectivity of HYL001. (A) Data for HYL001 toward a panel of 90 kinases (and 7 kinase variants) at 10 μM. 22 kinases inhibiting >50% (excluding those from Table S1) were subsequently tested at 3 μM and 300 nM. Visualized using TREE*spot*: interactions are depicted by red dots, with size proportional to inhibition of control ligand binding (shown if >65%). (B) Data for galunisertib and vactosertib at 10 μM toward the larger panel (*N* = 97). (C) Selectivity scores (fraction of wildtype kinases inhibited >65% or >90% at 10 μM compound concentration). *TK: tyrosine kinases; TKL: tyrosine kinase-like kinases; STE: serine/threonine kinases; CK1: casein kinase 1 family; AGC: serine/threonine kinases, regulated by secondary messengers such as cyclic AMP (PKA) or lipids (PKC); CAMK: Ca*^*2+*^*/calmodulin-dependent protein kinase family; and CMGC: primarily proline-directed serine/threonine kinases (CDKs, GSKs, MAP kinases, and CDK-like kinases).*

We also assessed the selectivity of these three inhibitors toward a panel of 97 wild-type and disease-relevant mutant kinases, distributed throughout various kinase families. An initial analysis at high concentration indicated possible interactions with 13 of the 90 kinases tested (>65% competitive binding inhibition by HYL001 at 10 μM) and with mutants of 2 additional kinases (Figure 2A). However, at a lower concentration (300 nM), the only off-target interaction left was with p38α.

Calculating the fraction of nonmutant target kinases with >65% inhibition at 10 μM, *S*(>65_10_) HYL001 scored 0.144. Its selectivity score for >90% inhibition *S*(>90_10_) was 0.1, which indicates a slightly lower selectivity compared to galunisertib but higher selectivity than vactosertib (Figure 2B,C).

### *In Vitro* Enzymatic Activity

ALK5 IC_50_ values for HYL001, galunisertib, and vactosertib were obtained in a kinase activity assay with recombinant proteins. Compared to galunisertib, the value for HYL001 of 177 nM constitutes an approximate 2.5-fold increase (Table 1), in accordance with the increase in binding affinity (Table S1). This IC_50_ of HYL001 is around 3.5-fold higher than that of vactosertib in this assay (Table 1). We also analyzed the kinase inhibition activity of HYL001 toward p38α (Table 1). HYL001 is a stronger inhibitor of p38α than galunisertib. Vactosertib, on the contrary, shows little activity toward this kinase, as it was specifically selected to not inhibit p38α.^18^

**Table 1:** *In Vitro* Kinase Inhibition of ALK5 and p38α and TGFβ Reporter Activity

	kinase inhibition IC_50_ (nM)			cellular inhibition TGFβ pathway IC_50_ (nM)		
	HYL001	galunisertib	vactosertib	HYL001	galunisertib	vactosertib
ALK5 (TGFBR1)	177	448	51	72	157	10
p38α	548	920	6893			

We next tested the three inhibitors in a cellular assay, using a luciferase-based reporter assay^20^ in HEK293T cells in the presence or absence of inhibitors (Table 1). Upon activation of the endogenous receptors by 5 ng/mL recombinant TGFβ, phosphorylation of the TGFBR1 triggers phosphorylation of the intracellular mediators SMAD2/3, and subsequent nuclear translocation with SMAD4. The SMAD2/3-SMAD4 complex binds to the TGFβ response element (12xCAGA) allowing the transcription of the Firefly luciferase reporter. In this assay, inhibition of TGFβ activity by HYL001 showed again an approximate 2-fold higher potency compared to galunisertib (Table 1). Vactosertib was more potent *in vitro* than HYL001 and galunisertib.

**Table 2:** Pharmacokinetics of HYL001 in Preclinical Species after Oral Administration^a^

	species	dose (mg/kg)	*T*_max_ (h)	*C*_max_ (ng/mL)	AUC_last_ (ng·h/mL)	AUC_inf_ (ng·h/mL)	*T*_1/2_ (h)	*V*_ss_/*F* (L/kg)	Cl/*F* (mL/min/kg)
HYL001	mice (♂ BALB/c)	71	0.25	2050	3520	4110	2.45	*66.7*	*288*
Gal^20^	mice (♀ CD-1)	75	0.5	3330	3110	NA	1.4	NA	*401.9*
Vacto^21^	mice (FVB/N)	10	0.5	625	724	752	3.26	*18*	*222*
HYL001	rats (♂ SD)	71	0.67	8542	21,575	22,007	4.8	*10.8*	*53.8*
Vacto^18^	rats (♀ SD)	10	NA	1620	NA	1426	2.5	NA	NA

a Data shown for galunisertib^20^ and vactosertib^18,21^ are from indicated publications. The coefficient of variation in mice ranged between 19 and 71%. *C*_max_; maximum plasma concentration; *T*_max_, time to *C*_max_; *V*_ss_/*F*, apparent volume of distribution at steady state; *F*, bioavailability; Cl/*F*, apparent total body clearance; and values in italics were estimated using mean plasma levels and NCA with Kinetica software v6.0. For Gal: Cl/F was estimated using AUC_last_;^20^ NA: not available.

### Pharmacological Characterization

To advance in the preclinical development of HYL001, we engaged in studies of *in vitro* characterization. Plasma protein binding (PPB) values of HYL001 were obtained for human, rat, and mouse plasma by equilibrium dialysis (90 < PPB < 95.2; Table S2). We also evaluated the metabolic stability of HYL001, galunisertib, and vactosertib human cryopreserved hepatocytes. In these assays, both HYL001 and vactosertib showed a similar pattern, with <50% parent compound remaining in human hepatocytes within 1 h of incubation (Table S3). Comparatively, galunisertib was more stable, with 76% remaining after 1 h of incubation.

We assessed the *in vivo* pharmacokinetic properties of HYL001 after a single oral administration (dose of 71 mg/kg) in BALB/c mice as well as in Sprague–Dawley (SD) Rats. Liquid chromatography–tandem mass spectrometry (LC/MS/MS) was used for the determination of HYL001 in plasma (Figure S2 and Table 2). Inspection of the plasma concentration–time profile for HYL001 revealed that the mean value of the maximum plasma concentration (*C*_max_) after oral dosing of 71 mg/kg was 2050 ± 1459 ng/mL in mice and 8542 ± 1220 ng/mL in rats. Peak plasma concentrations were observed at 0.25 h (mice, first time point tested) and 0.67 ± 0.29 h (rats), suggesting rapid absorption after oral dosing. The elimination kinetics of HYL001 demonstrated a moderate terminal half-life (*T*_1/2_): approximately 2.5 and 5 h in mice and rats, respectively. These values are higher than those observed for galunisertib and vactosertib.^18,20^

### *In Vitro* Safety Pharmacology

To identify potential adverse off-target drug interactions and derisk the development of HYL001 as a drug,^22^ we performed binding and enzymatic inhibition assays with a panel of key proteins (mainly cell receptors, neurotransmitter transporters, and ion channels). Binding causing >50% inhibition, considered to represent a potential safety concern, was not observed to any of the targets for HYL001 (Figure S3A), nor did HYL001 cause >50% inhibition of any of the tested enzymatic activities (Figure S3B).

Of note, inhibitory binding of the human Ether-à-go-go-Related Gene (hERG) potassium channel, which is not the case for HYL001 (Figure S3A), is indicative of cardiotoxicity. Given the history of TGFβ inhibitors with on-target toxicity,^14^ we further determined the activity of HYL001 toward hERG, more specifically using a patch-clamp assay.^23^ Unlike the reference compound E-4031 that blocks potassium channels of the hERG-type (exhibiting an IC50 of 37 nM, data not shown), HYL001 showed low inhibition at micromolar concentrations (Table S4). For comparison, the IC50 of vactosertib toward the hERG channel is 31.04 μM,^18^ indicating a lower cardiac toxicity risk for HYL001.

### Mutagenic Potential

The mutagenic potential of HYL001 was evaluated by the Ames test, assessing its ability to induce reverse mutations in the histidine operon of *Salmonella typhimurium* strains that allow for detection of both substitution mutations (TA100 and TA1535) and frameshift mutations (TA98 and TA1537). The mutagenic potential of HYL001 was tested at 6 concentrations (semilog 1–320 μg/mL) alone or in the presence of liver S9 fractions. No positive mutagenic responses were observed with any of the strains (Table S5).

### Preclinical Toxicity in Rodents

We performed exploratory preclinical toxicology studies with HYL001 in rats and mice, using vactosertib as a comparator drug.

#### Toxicology in SD Rats

**Figure 3:**
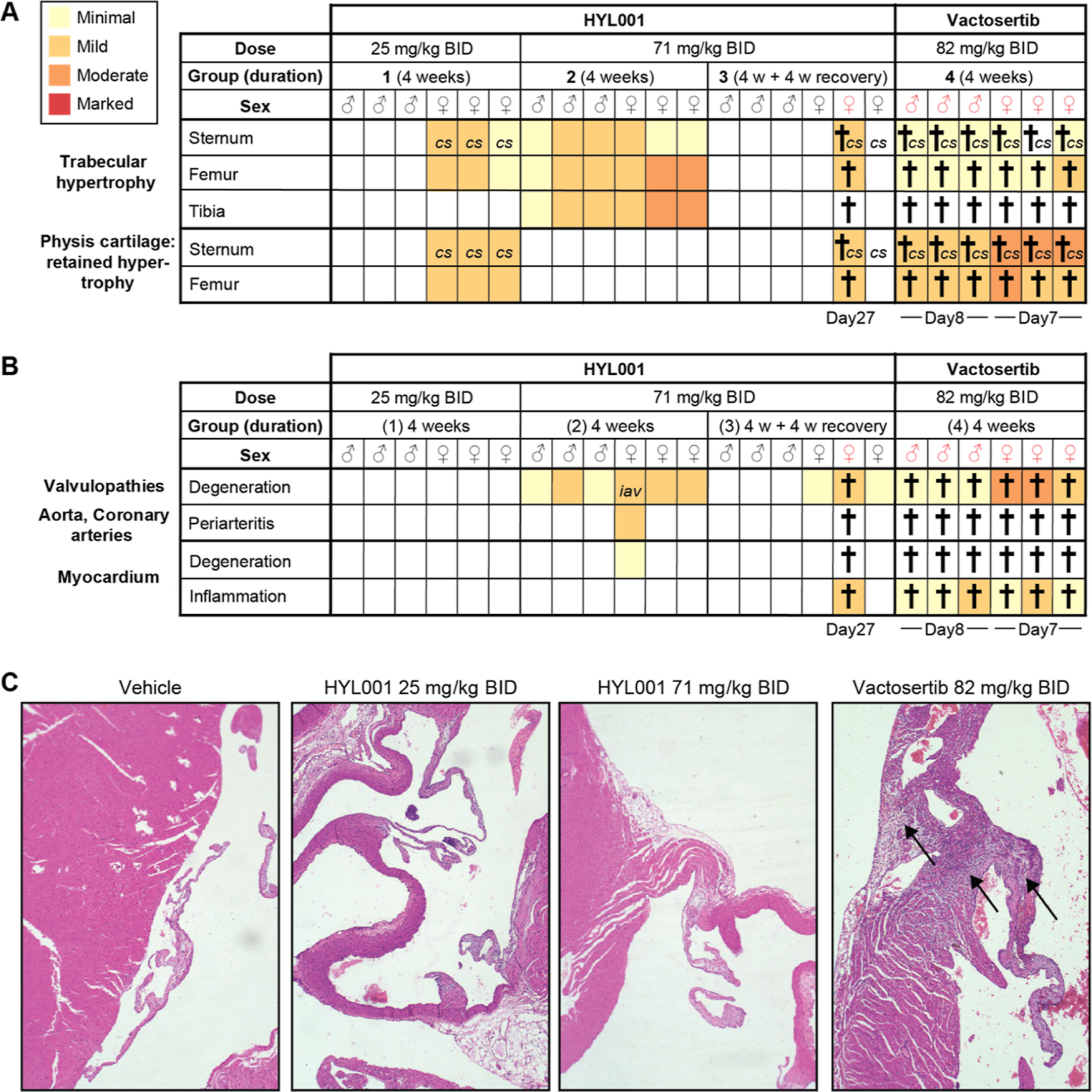
Rat toxicology study. (A,B) Overview of histopathological findings in bones (A) and heart (B) for four groups of rats with sex and (planned) duration indicated, as well as occurrence and time of animal death/clinical end point (†). The color scheme represents a 4-tier scale. Observations of curved sternum (*cs*) and chronic inflammation of the aortic valve (*iav*) are marked. (C) Examples of rat heart valves and adjacent myocardium in a healthy control or after indicated treatment. Arrows indicate degeneration of the valve and chronic inflammation of adjacent myocardium. All images at 10× magnification. *BID: bis-in-die; twice daily*.

Four groups (*n* = 6) were studied, three with HYL001 at two dose levels and the fourth with vactosertib at an equimolar dose compared to groups 2 and 3 (Figure 3A). In 6 vehicle control-treated rats, no abnormalities were detected (not shown). There were no HYL001-related effects on body weight (gain), feed consumption, hematology, and clinical chemistry analytes in rats of both sexes treated orally with HYL001 at either dose level. Also, no clinical findings were noted at the lower. However, 3/6 female group 3 animals treated with 71 mg/kg BID (twice daily) developed signs of lethargy, tachycardia, and protrusion of the sternum from day 8 of treatment. One of these females died on day 27. The 2 remaining females from this group recovered from lethargy during the off-treatment period. Males in group 3 only showed rough hair coats during the last 2 weeks of treatment, from which they soon recovered. 17/18 (94%) rats treated with HYL001 survived until the end of the study.

In contrast, in vactosertib-treated rats (group 4), animals gained signs of lethargy, protruded sternums, and mildly reduced locomotor activity (in males); and of lethargy, tachycardia, mildly reduced locomotor activity, rough coat, piloerection, and protruded sternum (in females), all within the first week of treatment. Strikingly, all female rats were found dead on day 7 and all male rats had to be sacrificed on day 8 (Figure 3A). Given the previously reported on-target side-effects of TGFβ inhibitors,^14,18,24,25^ detailed histopathological analysis focused on bones, heart, and gastrointestinal system was carried out in all groups.

##### Bones

Some female rats (HYL001) and all vactosertib-treated animals showed a curved sternum at the death or end point. Increased bone formation in trabeculae (trabecular hypertrophy) was observed in the femur, tibia, and sternum, in both sexes and with both compounds (Figure 3A). This effect appeared to be reversible, as it was not found after the 4 week recovery period in group 3. Retained hypertrophied cartilage physis was identified in the sternum and femur of all vactosertib-treated animals (mild–moderate) and in a few female HYL001-treated animals (mild). The latter abnormalities have been described before for ALK5 inhibitors^13,25^ yet are predicted to have low impact in adult humans, since their growth plates are expected to be closed. Also, these effects did not prevent galunisertib or vactosertib from entering clinical trials.

##### Heart

The major safety concern regarding ALK5 inhibitors is the on-target cardiac toxicity observed in prior preclinical studies, which included valvulopathies (degenerative and inflammatory valvular lesions), myocardial degeneration/necrosis, hemorrhage, or mixed cell infiltrates in the myocardium, necrosis with inflammation of coronary arteries, and mixed cell infiltrates in the atrium.^14,18,26^ We observed minimal to mild degeneration of heart valves in both male and female HYL001-treated rats at terminal examination after 4 weeks of repeated dosing in group 2 as well as the female from group 3 that died on-treatment (Figure 3B,C). This consisted of myxomatous expansion of the stroma or stromal proliferation. However, the heart valves of HYL001-treated rats were almost normal after a 4 week recovery. Relatedly, an increase in absolute and relative weight of the heart was found in female HYL001-treated rats after 4 weeks of treatment (group 2), yet this was much reduced after 4 weeks of recovery (group 3). With vactosertib, moderate degeneration of valves was already observed after 7–8 days of treatment when the rats died or had to be sacrificed. Both the curved sternum and increased weight of the heart have been reported previously for galunisertib^24^ that was later safely used in patients. Furthermore, vactosertib is also being used in patients.

Inflammation of the aortic valve and periarteritis in the aorta and coronary arteries were found in one female rat under HYL001 treatment (group 2), yet in none of the rats of the recovery group. These abnormalities were not observed in vactosertib treated animals at their (unintended) early end points. However, at that time, chronic inflammation of the myocardium was observed in all 6 vactosertib-treated animals, as well as in the female rat treated with HYL001 that was found dead (Figure 3B,C). Only one female rat in group 2 showed minimal degeneration of the myocardium, characterized by infiltration of mononuclear cells (predominantly lymphocytes and macrophages) and necrosis of cardiomyocytes. This was not observed in the recovery group (Figure 3B). In fact, cardiac toxicity observations were absent or minimal in the 5 remaining rats of this recovery group, suggesting reversibility. In addition, no heart abnormalities were observed in low-dose group 1 (Figure 3B). In this group, mild histopathological findings were observed in the sternum and femurs of female rats only. As mentioned above, these are predicted to have a small impact on adult patient populations. For male rates, the no-observed-adverse-effect level of HYL001 was established at the dose of 25 mg/kg BID, similar to the reported NOAEL of vactosertib.^18^

##### Gastrointestinal Tract

No microscopic findings were observed along the gastrointestinal tract, except minimal hyperplasia of the nonglandular stomach and chronic inflammation observed in one female rat under vactosertib treatment.

#### Toxicology in Balb/C Mice

**Figure 4:**
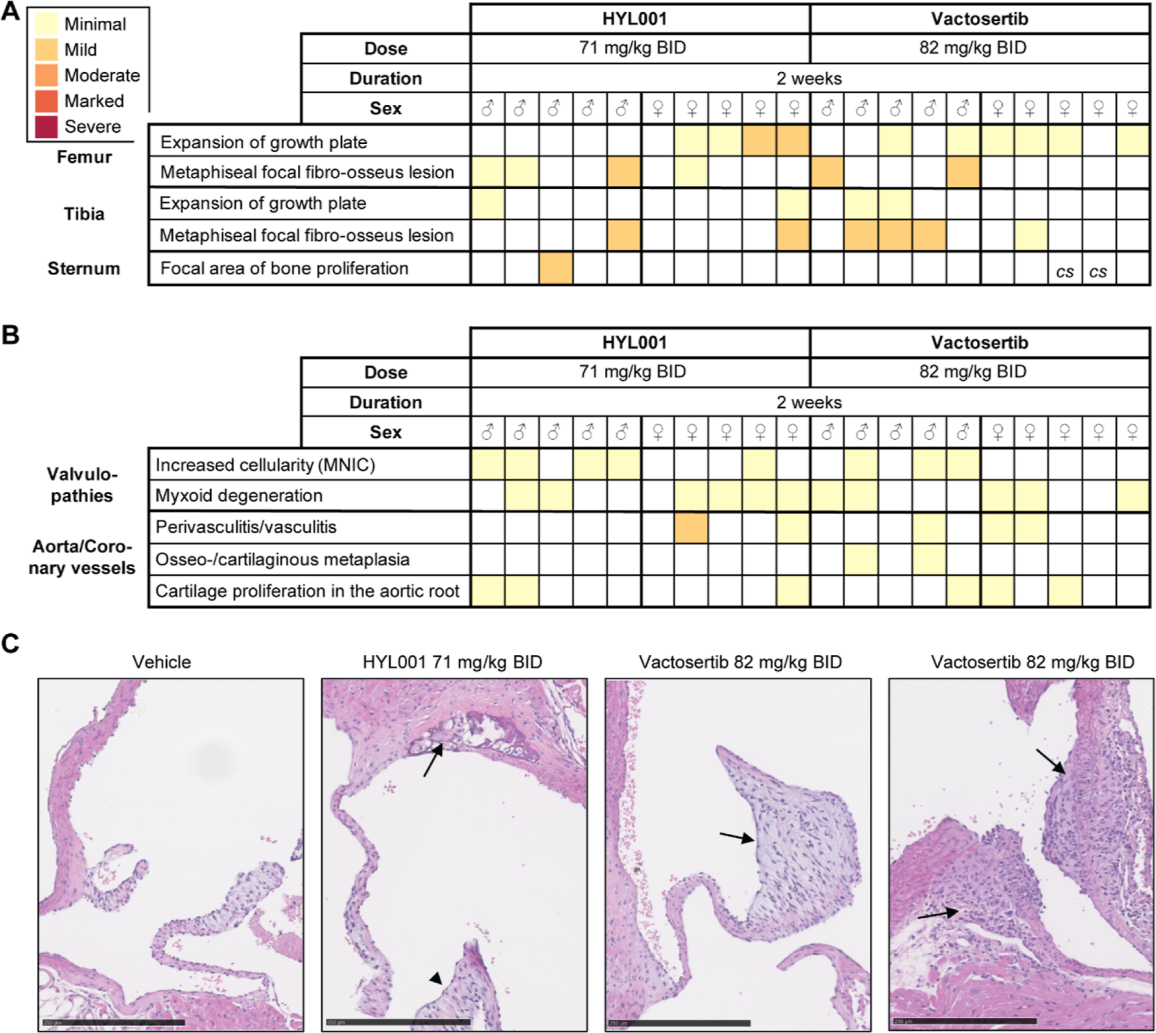
Mouse toxicology study. (A,B) Overview of histopathological findings in bones (A) and heart (B) for indicated sex and treatment. The color scheme represents a 5-tier scale. Observations of curved sternum (*cs*) are indicated. *MNIC:* mononuclear immune cell. (C) Representative examples of a semilunar valve from a vehicle control-treated mouse (left); a semilunar valve with myxoid degeneration (arrowhead) and cartilaginous metaplasia in the root of the aorta in a HYL001-treated animal (arrow; middle left); a semilunar valve with myxoid degeneration and increase in cellularity from a vactosertib-treated mouse (arrow; middle right); and focal mononuclear perivasculitis/vasculitis in coronary vessels in a vactosertib treated mouse (arrow, right). All images at 20× magnification, scale bar: 250 μm.

In comparison to that in rats, toxicity of TGFβ receptor-1 inhibitors in mice was less severe (Figure 4). No mortality or signs of morbidity were observed for HYL001 or vactosertib at equimolar dose. Specifically, there were no compound-related effects on body weight, feed consumption, hematology, and clinical chemistry analytes in mice of either sex, except for a slight increase in neutrophil count for vactosertib-treated males. Histopathology examination in bones found relatively mild phenotypes (Figure 4A).

##### Bones

Minimal–mild signs of proliferation of fibro-osseous tissue in the metaphysis (bone hypertrophy) were observed in mice with either compound, especially in the femur and tibia. Despite two cases of macroscopically curved sternum, histopathological observations were almost lacking there (Figure 4A).

##### Heart

There were no alterations in any of the myocardia. However, similar to the rats, treated mice exhibited some minimal to mild myxoid degeneration of heart valves, which were also observed in 2/10 vehicle control-treated mice (not shown), or an increase in cellularity mainly from mononuclear immune cells (Figure 4B,C). Mice under both treatments presented focal mononuclear perivasculitis or vasculitis in coronary vessels. Some mice under both treatments also showed associated incipient osseous-cartilaginous metaplasia at the root of the aorta or coronary vessels. This phenotype was more pronounced in vactosertib treated mice.

##### Gastrointestinal Tract

No observations were made except for slight inflammatory infiltrates, mainly with mononuclear cells, in some female livers (2/5 mice treated with HYL001 and 4/5 for vactosertib).

Taken together, female animals exhibited more treatment-related toxicities than males, and rats were more sensitive than mice. Importantly, HYL001 toxicity in SD rats appears to be milder than that of an equimolarly dosed compound currently in clinical trials.

### *In Vivo* HYL001 Efficacy as Immunotherapy against CRC Metastases

We next assessed the *in vivo* efficacy of HYL001 in our model of pMMR/MSS CRC.^5^ As reported, we generated a genetic mouse model bearing mutations in key CRC pathways (WNT, P53, MAPK, and TGFβ) and derived mouse tumor organoids (MTOs) from invasive adenocarcinomas as well as liver metastases generated in quadruple compound mice. Both the genetic model and mice implanted with MTOs developed intestinal tumors and metastatic liver lesions that reproduced several key features of human poor prognosis CMS4-like CRC, including a stroma-rich, TGFβ-activated TME and low levels of T cell infiltrations.^5^ Subsequently, this MTO-based system has proven a powerful preclinical model, both to dissect epithelial tumor cell heterogeneity that is representative for human cancer cell states^27,28^ and to further dissect the TME for additional mechanisms of (dys)regulation of cancer immunity.^29−32^

**Figure 5:**
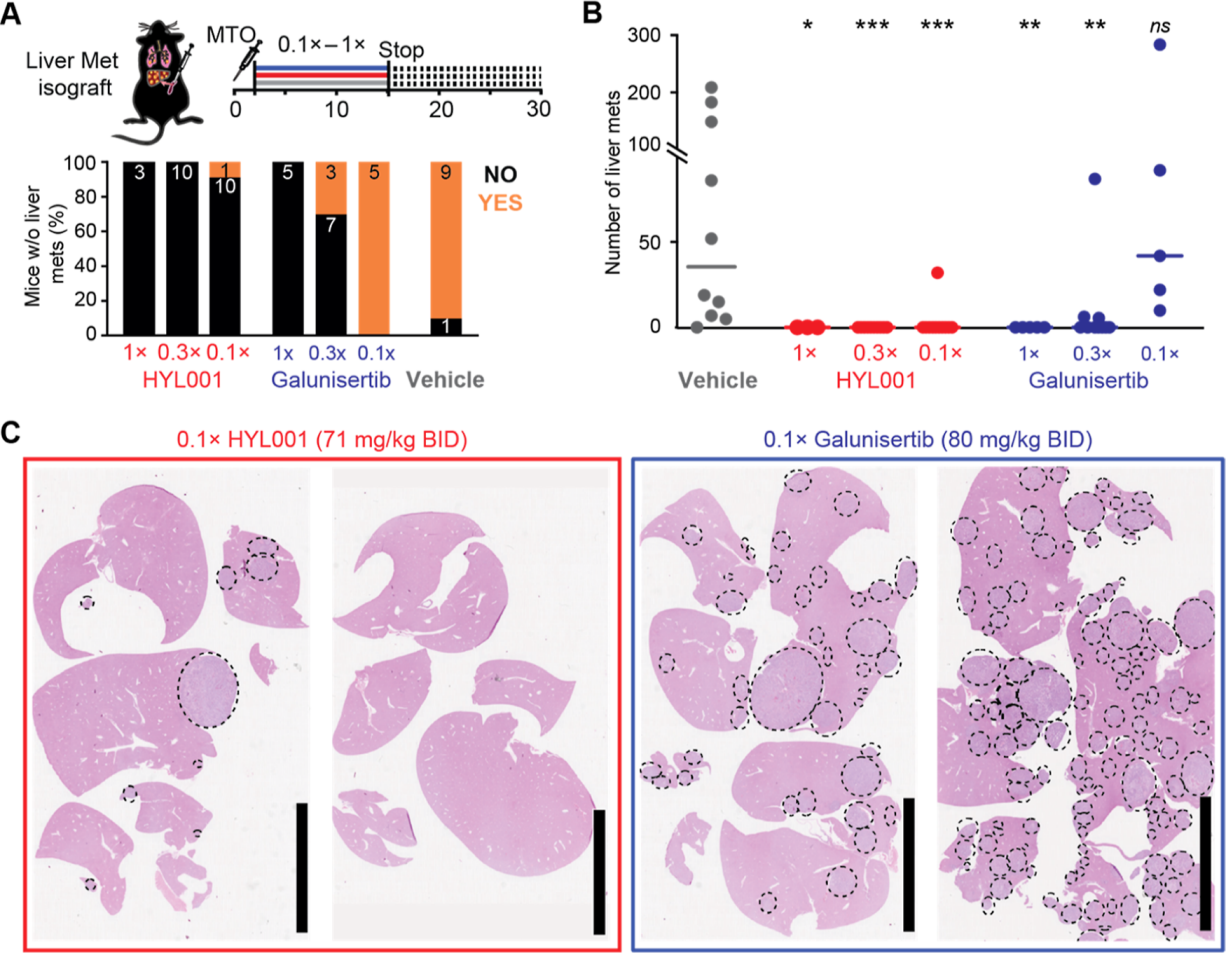
HYL001 prevents metastasis formation *in vivo*. (A) Experimental scheme, including injection of MTOs. Experimental end point was at day 28, 2 weeks after treatment stop. Below: the number of mice treated that had (in orange) or did not have (in black) detectable liver metastases.^5^ (B) Liver metastases (Mets) were counted at end points; each point represents one mouse. Nonparametric Mann–Whitney *U* test, two-tailed. ns: *P* > 0.05; *P < 0.05; **P < 0.01; ***P < 0.001; (C) representative images of H & E whole slides containing liver sections from mice treated with TGFβ inhibitors; dashed line circles delimit metastases. 1×, efficacious dose for galunisertib established in ref (5): 800 mg/kg BID, and molar equivalent for HYL001 (710 mg/kg BID). 0.3× and 0.1×: dilutions thereof. Bars, 10 mm.

We first assayed the capacity of HYL001 to inhibit liver metastasis formation as a monotherapy. In this model, we inoculated MTOs derived from liver metastases into the portal vein (by intrasplenic or direct intravenous injection) to model their becoming trapped in hepatic sinusoids and, depending on their metastatic capacity, the initiation of liver nodules. We had previously demonstrated that galunisertib can prevent the formation of liver metastases using this model, albeit at a very high dose (720–800 mg/kg BID),^5^ which is likely far above clinically relevant levels. Normalizing for the 800 mg/kg BID galunisertib dose, we tested a dose range of 0.03 × −0.9 × mol equiv (21–637 mg/kg BID) of HYL001. Remarkably, 0.1 × mol equiv (71 mg/kg BID) of HYL001 was sufficient to prevent metastasis formation in 10 out of 11 animals. All treated animals developed metastases with galunisertib at this 0.1× dose level (80 mg/kg BID) (Figure 5).

Furthermore, treatment of mice that had already established liver metastases with HYL001 resulted in a marked decrease in protein expression of relevant TGFβ targets, including phospho-SMAD2, CTHRC1, and Caldesmon^9^ (Figure S4). Despite the role of TGFβ blockade in preventing liver metastatic initiation in our mouse model, which we showed to involve T cell-mediated anticancer immunity,^5^ established metastatic CRC was not cured by this treatment in our model. Moreover, these mice were resistant to immune checkpoint therapy like human patients with pMMR/MSS CRC are in the clinic.^5,33^ However, we showed that TGFβ inhibition mediated by galunisertib (at high concentrations) combined with immune checkpoint therapy could overcome such resistance and synergize with immunotherapies to cure established metastases.^5^ For these experiments, we used the 1× galunisertib dosage (800 mg/kg BID).^5^

**Figure 6:**
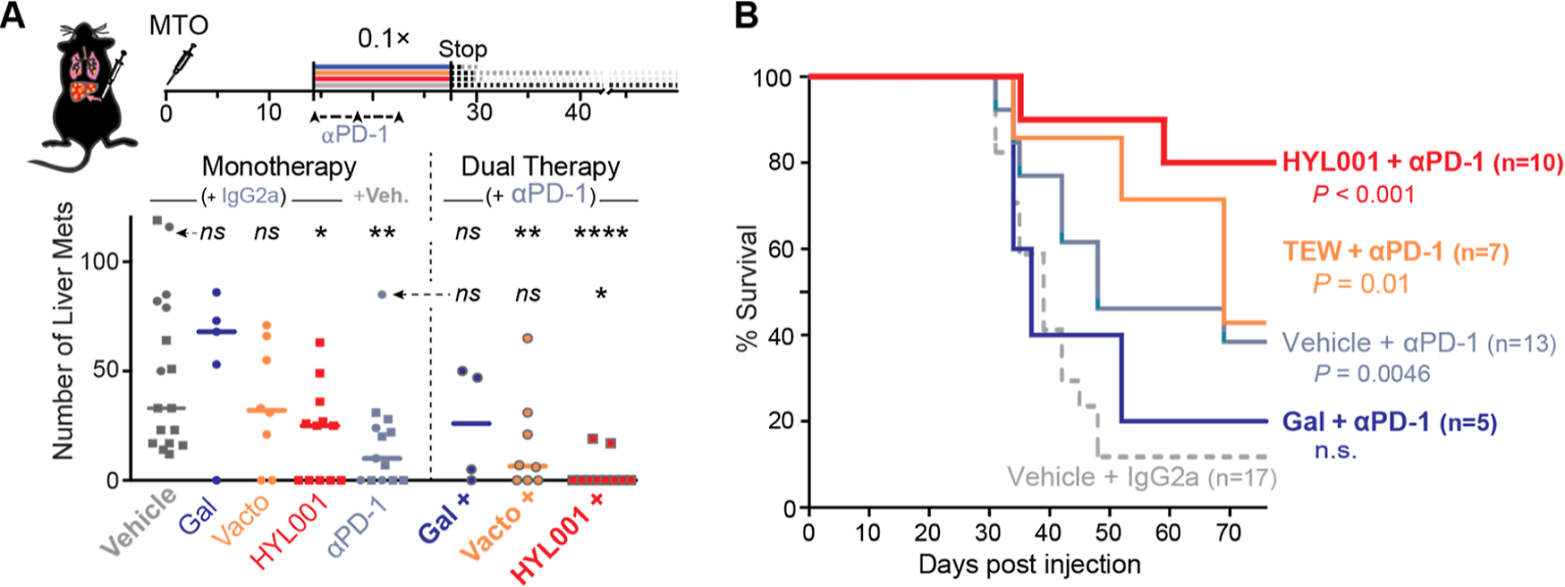
HYL001 efficacy in dual immunotherapy against established CRC liver metastases. (A) Overview of experimental timing: dual treatment between days 14 and 28 after MTO injection (TGFβ inhibitor doses were all 0.1 ×); follow-up until humane end point. Below: numbers of countable liver lesions after sacrifice. Statistical comparisons to the vehicle + IgG2a control (above) or the vehicle+αPD1 condition (below) by two-tailed Mann–Whitney *U* test; *ns*: *P* ≥ 0.05; *P < 0.05; **P < 0.01; ***P < 0.001; ****P < 0.0001. (B) Kaplan–Meier curves indicate the survival (until humane end point) of mice under each treatment; *P* values were computed by the Mantel–Cox test (Log-rank).

Encouraged by the increased potency observed *in vitro* and in the inhibition of liver metastatic initiation *in vivo*, we evaluated the ability of a relatively low HYL001 dose to help overcome established liver metastases when combined with immune checkpoint inhibition. In this experimental set up, a low number of MTO cells (25K) was injected in the portal vein (leaving the spleen unaltered), and liver metastases were allowed to develop and grow for 15 days before treatment with TGFβ inhibitors was initiated. This was done in combination with 3–4 intraperitoneal (IP) injections of immune-checkpoint inhibition therapy (anti-PD1 or isotype control antibody), spaced 2–3 days (Figure 6A,B). In accordance with our previous results,^5^ immune checkpoint therapy alone was not efficacious to cure liver-established metastases, and neither were the three TGFβ inhibitors as monotherapy (80, 82, and 71 mg/kg BID equimolar 0.1× doses for galunisertib, vactosertib, and HYL001, respectively; Figure 6A). In dual therapy, HYL001 exhibited the highest efficacy curing up to 80% of mice (8/10) at the end point (surviving for 2.5 months after injection, the study end point) and a marked overall reduction of tumor burden in numbers of liver nodules (Figure 6A,B).

## Discussion and Conclusions

Despite long-standing and active interest in therapeutically targeting the TGFβ pathway, drug development has not yet led to successful treatments due to low clinical efficacy and/or high toxicity in humans.^6,13,34^ Here, we present HYL001, a TGFBR1/ALK5 inhibitor that has increased *in vitro* and *in vivo* potency at relatively low doses and has favorable preclinical safety characteristics. We show that HYL001, while structurally related to galunisertib, is functionally superior in eliciting T cell-mediated eradication of CRC liver metastasis in mice. In direct comparison with vactosertib (TEW-7197), an unrelated second-generation inhibitor that is in clinical trials for solid cancers including CRC, HYL001 has similar or better efficacy at an equimolar dose range in our mouse experiments, albeit a safer toxicity profile in mice and rats. Therefore, HYL001 is a promising inhibitor of TGFβ signaling with clear preclinical benefit, offering an economical advantage for *in vivo* research, that also warrants clinical testing. Indeed, in our view, the focus should be foremost on a treatment that is safe and feasible to test for clinical efficacy. Nevertheless, it would be interesting to study HYL001 in other sophisticated animal models for, *e.g.*, triple-negative breast or lung cancer.

Whereas the increased efficacy of HYL001 may arise solely from a distinction in on-target affinity due to the structural difference with galunisertib, the higher degree of improvement *in vivo* over *in vitro* (∼9-fold *vs* ∼2.5-fold) suggests an additional effect. We investigated PPB, hepatic stability, and PK in this study. The reduced metabolic stability in hepatocyte culture, compared to galunisertib, may be related to the relatively low (∼91%) PPB value for HYL001 in humans, suggesting a potentially higher unbound fraction. On the other hand, this may also be associated with elevated drug–target interaction, although *in vivo* drug kinetics are rather more complex than can be captured by a PPB value.^35^ Toward a more comprehensive understanding thereof, our PK studies show good gastrointestinal absorption and indicate clearance values for HYL001 that are slower than both galunisertib and vactosertib. Therefore, the increased efficacy *in vivo* might be attributed to the sum of favorable traits in a number of variables.

Measuring *in vitro* affinity for ALK5, vactosertib showed the lowest IC_50_, galunisertib showed the highest, and HYL001 has an intermediate value. We subsequently performed a selectivity screen using an *in vitro* competitive binding assay and observed binding preference, besides for ALK5, toward ALK4/ACVR1B for all 3 compounds. We further found that galunisertib and HYL001 are moderately selective with only a few differences, with HYL001 additionally showing >85% competitive binding to ABL-1, CSNK1G2, and p38α in our assay. Vactosertib is clearly less selective, showing >85% competitive binding to an additional set of targets that includes ERBB2, FGFR2/3, KIT, MEK1/2, PDGFRA/B, SRC, and VEGFR2. Whereas these distinct on- and off-target affinity profiles might translate into varying *in vivo* efficacy, we did not observe strong differences there. As poor selectivity for multitarget kinases has been hypothesized to lead to a concentration-dependent broadening of the inhibitory spectrum,^36^ the lack of evident differences together with the relatively low doses used in our current study indicate that the immuno-oncological effect of inhibitors is indeed on-target.

Nevertheless, it is possible that the lower selectivity for vactosertib may be related to its comparatively elevated toxicity. Vactosertib is being given to patients with cancer at a 200–600 mg daily dose.^37−39^ Assuming an average weight of 60 kg, this would represent a 3–10 mg/kg dose, which in turn can be converted (using a body weight–surface area scaling ratio of 12.3)^40^ to an equivalent of a 41–123 mg/kg daily dosage in mice. This is just below what we tested here, and our 0.1× dose (164 mg/kg daily, or a human equivalent of 800 mg) would therefore be expected to risk cardiac or other toxicities. Clinical data however suggest that these levels are sufficiently safe,^37−39^ suggesting differences between toxicology findings in rodents and in patients. Indeed, we have shown relevant differences in toxicity also exist between rodents. In comparison, galunisertib required a ∼ 9-fold higher dosing range (in human equivalents >7 g daily) to be efficacious in our CRC model, dose levels that are unfeasible for human translation. The fact that the maximum-tolerated dose used in clinical trials is lower than what appears to be required for galunisertib may explain the lack of therapeutic window for this first-generation compound.^13^ HYL001 at the dose levels used here (2 × 71 mg/kg daily in mice; human equivalent: 692 mg/day) performed very well, with an even more favorable toxicity profile than vactosertib in either rodent model.

In our preclinical efficacy study, we used MTOs introduced into portal vein circulation of immunocompetent (syngeneic) mice to induce the formation of liver metastases.^5^ We described the benefit of using organoids over available cell lines previously and linked the characterization of our model, as mismatch-repair proficient, microsatellite-stable invasive adenocarcinoma with human-like histopathology, to the majority of cases of mCRC.^5^ These patients are not likely to respond to ICI monotherapy^41^ but may benefit from combinatorial immunotherapies such as the added blockade of TGFβ signaling.^3,5,6^ Indeed, mounting evidence connects this pathway to dominant changes in the TME and suppression of immunity in particular,^4^ yet it simultaneously emphasizes the contextual, pleiotropic functions of TGFβ, some of which are linked to severe toxicities in the context of systemic inhibition. Besides leading to several alternative therapeutic strategies, including blockade of upstream activation, targeted ligand traps, and singling out individual isoforms,^6,7,13^ there remains potential for a small molecule TGFβ receptor kinase inhibitor with relatively low toxicity and retained potency. In comparison to, for example, fusion protein TGFB ligand traps, having a 1:1 stoichiometry and high production cost, a compound (with a chemical handle) can be fine-tuned to enhance efficacy and tolerability and should be more cost-effective. Moreover, preclinical data for some upcoming inhibitors (increasingly biologicals, *e.g.* SRK-181 and bintrafusp alfa) often exerted only partial therapeutic responses in experimental models,^26,42^ while effectively depleting plasma TGFβ levels,^43^ potentially indicating trap quenching before reaching the TME.

In conclusion, we have synthesized a new small molecular inhibitor targeting TGFBR1 activity that has the required potency to inhibit a TGFβ-rich stroma and synergize with immunotherapies in advanced models of poor prognosis of CRC. HYL001 provides superior potency compared to galunisertib at 9-fold lower dosing, without added toxicity, and compares favorably to vactosertib, another second-generation compound. The combined data we present here support a clinical study to confirm safety and feasibility for prolonged dosing in patients. Subsequently, HYL001, or functional derivatives, taking advantage of its “handle”, should be tested to treat metastatic cancers that thrive in a highly TGFβ-mediated immunosuppressive microenvironment.

## Experimental Section

### Synthesis of Compounds

All new compounds were chemically synthesized, purified by chromatography, and characterized by ^1^H NMR, ^13^C NMR, IR, and HRMS. Compounds were of a purity ≥95%, as determined by HRMS and ^13^C NMR spectroscopy, or by HPLC/MS. NMR spectra were recorded at 23 °C on a Varian Mercury 400 or Varian 500 apparatus. ^1^H and ^13^C NMR spectra were referenced either to relative internal TMS or to residual solvent peaks. IR spectra were recorded in a Thermo Nicolet Nexus FT-IR apparatus. Melting points were determined using a Büchi M-540 apparatus. HRMS were recorded in a LTQ-FT Ultra (Thermo Scientific) using the nanoelectrospray technique. HPLC chromatography was performed on Hewlett-Packard 1050 equipment with UV detection using a Kinetix EVO C18 50 mm × 4.6 mm, 2.6 μm column [standard gradient: 10 mM NH_4_CO_3_/MeCN (95:5) – (0:100)]. All reactions were carried out under an inert atmosphere (N_2_) unless otherwise stated.

The following compounds were prepared following standard or reported procedures: galunisertib,^8,44^ vactosertib,^18,45^ 6-methoxy-4-methylquinoline (**3a**),^46,47^ 6-bromo-4-methylquinoline (**3b**),^16^ and 1-aminopyrrolidin-2-one tosylate salt.^44^

#### 2-(6-Methoxyquinolin-4-yl)-1-(6-methylpyridin-2-yl)ethan-1-one (**4a**)

A solution of methyl 6-methylpicolinate (5.05 g, 33.4 mmol) in 15 mL of dry THF was stirred under a N_2_ atm. for 1 h in the presence of 0.5 g of 3 Å molecular sieves. 6-Methoxy-4-methylquinoline (**3a**, previously dried under Dean–Stark conditions; 2.90 g, 16.7 mmol) was dissolved in 20 mL of anh THF under N_2_ atm, and the resulting solution was cooled to −40 °C. A 2 M solution of LDA in THF (19.2 mL, 38.4 mmol) was added dropwise over the quinoline solution, and the resulting dark green suspension was stirred for 30 min at −40 °C. After this time, the anion and the previously prepared ester solution were cooled down to −78 °C, and the anion was added over the ester. The resulting mixture was left to reach room temperature and stir overnight. Then, 100 mL of a saturated solution of NH_4_Cl was added, and the aqueous layer was extracted with EtOAc (3 × 100 mL). The combined organic extracts were dried over anh MgSO_4_, concentrated *in vacuo*, and purified by column chromatography (eluted with a gradient of hexanes and EtOAc from 100:0 to 0:100). The desired product (**4a**) was isolated as an orange oil (3.37 g, 69%). The spectroscopic properties were identical to the ones described;^48^^1^H NMR (400 MHz, CDCl_3_) δ 8.70 (m, 1H), 8.04–7.95 (m, 1H), 7.84 (d, *J* = 7.6 Hz, 1H), 7.71 (t, *J* = 7.7 Hz, 1H), 7.43–7.31 (m, 4H), 4.95 (s, 2H), 3.83 (s, 3H), 2.67 (s, 3H) ppm. ^13^C NMR (101 MHz, CDCl_3_) δ: 198.2, 158.1, 157.8, 152.2, 147.5, 144.5, 140.4, 137.2, 131.5, 128.9, 127.2, 123.8, 121.5, 119.6, 102.5, 55.4, 40.8, 24.4 ppm. IR (film): 3377, 3065, 2969, 2932, 2827, 1693, 1624, 1577, 1520, 1242, 1230 cm^–1^. HRMS (ESI): *m*/*z* [M + H]^+^ calcd for C_18_H_16_N_2_O_2_, 292.1212; found, 292.1208.

#### 2-(6-Bromoquinolin-4-yl)-1-(6-methylpyridin-2-yl)ethan-1-one (**4b**)

6-Bromo-4-methyl-quinoline (**3b**, 18.1 g, 81.7 mmol) was charged in a flask and purged with N_2_. Anh THF (345 mL) was added, and the resulting solution was cooled to −20 °C. To this solution, NaHMDS 2 M (123 mL, 245 mmol) was slowly added. The resulting mixture was stirred for 3 h at −20 °C to form the corresponding anion. Under anhydrous conditions, a solution of methyl-6-methylpicolinate (13.3 g, 98 mmol) in anh THF (40 mL) was added dropwise to the anion solution *via cannula*. The resulting mixture was stirred at −20 °C for 18 h. After the reaction was completed, THF was concentrated until 10% of the initial volume was left. EtOAc (300 mL) and a saturated solution of NH_4_Cl (600 mL) were added, and the biphasic mixture was stirred until all the solid was dissolved. Phases were separated, and the aqueous phase was extracted with more EtOAc (2 × 300 mL). The resulting organic extracts were dried over MgSO_4_ and filtered, and the solvent was removed under reduced pressure.

MeOH (675 mL) was added, and the mixture was stirred at rt 16 h. The resulting suspension was filtered, and the yellow residue was rinsed with more MeOH. Solvent was removed under reduced pressure to afford a red solid (24.2 g, 87%) that was directly used in the next reaction. The spectroscopic properties were identical to the ones described;^16^^1^H NMR (400 MHz, CDCl_3_) δ 8.86 (d, *J* = 4.6 Hz, 1H), 8.41 (d, *J* = 2.2 Hz, 1H), 8.15–8.08 (m, 1H), 7.90–7.85 (m, 1H), 7.81 (dd, *J* = 9.0, 2.1 Hz, 1H), 7.75 (t, *J* = 7.7 Hz, 1H), 7.52 (d, *J* = 4.6 Hz, 1H), 7.44–7.37 (m, 1H), 5.00 (s, 2H), 2.70 (s, 3H) ppm. ^13^C NMR (101 MHz, CDCl_3_) δ: 197.9, 158.4, 152.0, 150.5, 147.2, 141.3, 137.4, 132.8, 132.0, 129.4, 127.6, 127.1, 124.1, 120.9, 119.8, 40.5, 24.6 ppm.

#### 6-Methoxy-4-(2-(6-methylpyridin-2-yl)-5,6-dihydro-4*H*-pyrrolo[1,2-*b*]pyrazol-3-yl) Quinoline (**5a**)

2-(6-Methoxyquinolin-4-yl)-1-(6-methylpyridin-2-yl)ethan-1-one (**4a**, 3.37 g, 11.5 mmol) and 1-aminopyrrolidin-2-one tosylate salt (3.77 g, 13.8 mmol) were dissolved in a mixture of toluene (44 mL), DMF (10 mL), and 2,6-lutidine (3.4 mL). The resulting solution was heated up in a Dean–Stark for 16 h. Then, cesium carbonate (7.52 g, 23.1 mmol) was added, and the suspension was stirred 16 h under Dean–Stark conditions. Water (150 mL) was added to the reaction, and the aqueous layer was extracted with EtOAc (3 × 150 mL), dried over anhydrous MgSO_4_, and concentrated *in vacuo*. The crude product was purified by column chromatography (eluted with a gradient of hexane and EtOAc from 50:50 to 0:100) to yield 3.29 g (80%) of **5a**. The spectroscopic properties were identical to the ones described;^48^^1^H NMR (400 MHz, CDCl_3_) δ 8.74 (d, *J* = 4.4 Hz, 1H), 7.98 (d, *J* = 9.2 Hz, 1H), 7.35–7.20 (m, 4H), 6.96–6.85 (m, 3H), 4.37 (t, *J* = 7.2 Hz, 2H), 3.54 (s, 3H), 2.91 (br s, 2H), 2.75–2.65 (m, 2H), 2.38 (s, 3H) ppm. ^13^C NMR (101 MHz, CDCl_3_) δ: 158.6, 157.7, 153.7, 151.8, 147.7, 146.7, 144.9, 139.9, 136.4, 131.2, 128.3, 122.4, 122.2, 121.9, 119.5, 110.5, 104.0, 55.5, 48.5, 26.2, 24.6, 23.3 ppm.

HRMS (ESI): *m*/*z* [M + H]^+^ calcd for C_22_H_20_N_4_O, 356.1637; found, 356.1639.

#### 6-Bromo-4-(2-(6-methylpyridin-2-yl)-5,6-dihydro-4*H*-pyrrolo[1,2-*b*]pyrazol-3-yl) Quinoline (**5b**)

To a stirred solution of 2-(6-bromoquinolin-4-yl)-1-(6-methylpyridin-2-yl)ethan-1-one (**4b**, 19.8 g, 58.0 mmol) in DMF (60 mL), toluene (268 mL) and 2,6-lutidine (20 mL) at rt was added 1-aminopyrrolidin-2-one tosylate salt (18.9 g, 69.6 mmol). The reaction was heated to reflux under Dean–Stark conditions until most of the starting material had been consumed, as indicated by TLC. The mixture was cooled to rt, cesium carbonate (37.8 g, 116 mmol) was added, and the mixture heated to reflux. The reaction was monitored by TLC until all of the reaction intermediate was consumed. Then, toluene was distilled until the reaction mixture reached 145 °C and then cooled to rt. Water was added (630 mL), and the mixture was stirred at 0 °C for 2 h. The precipitate was filtered and washed with more water to obtain a brown solid that was purified twice through a silica column (eluent: EtOAc/MeOH from 0 to 5%) to obtain 5b as a pale-brown solid (16.1 g, 68%). The spectroscopic properties were identical to the ones described;^16^^1^H NMR (400 MHz, CDCl_3_) δ 8.85 (d, *J* = 4.4 Hz, 1H), 7.98 (d, *J* = 8.9 Hz, 1H), 7.92 (d, *J* = 2.2 Hz, 1H), 7.71 (dd, *J* = 8.9, 2.2 Hz, 1H), 7.35 (t, *J* = 7.7 Hz, 1H), 7.31 (d, *J* = 4.4 Hz, 1H), 7.11 (d, *J* = 7.8 Hz, 1H), 6.92 (d, *J* = 7.6 Hz, 1H), 4.37 (t, *J* = 7.2 Hz, 2H), 2.90–2.84 (m, 2H), 2.75–2.66 (m, 2H), 2.24 (s, 3H) ppm. ^13^C NMR (101 MHz, CDCl_3_) δ: 158.2, 153.6, 153.2, 151.3, 150.2, 147.1, 146.7, 140.9, 136.3, 132.5, 131.3, 128.7, 128.7, 123.0, 121.8, 120.2, 118.9, 48.3, 26.0, 24.2, 23.2 ppm.

#### 4-(2-(6-Methylpyridin-2-yl)-5,6-dihydro-4*H*-pyrrolo[1,2-*b*]pyrazol-3-yl)quinolin-6-ol (HYL001)

*Route a.* From **5a**: A suspension of NaH (60% dispersion in oil, 6.41 g, 160 mmol) was added to 60 mL of anh. DMF was prepared under N_2_ atm., and 38.4 mL (160 mmol) of dodecanethiol were added to form a thick foam. Then, a solution of 11.4 mg (32.1 mmol) of 6-methoxy-4-(2-(6-methylpyridin-2-yl)-5,6-dihydro-4*H*-pyrrolo[1,2-*b*]pyrazol-3-yl)quinoline (**5a**) in 90 mL of anh DMF was added *via* cannula, and the mixture was heated up to 150 °C and stirred for 30 min. Then, the reaction was cooled to rt, diluted with EtOAc (100 mL), and extracted twice with NaOH (1 M, 100 mL). The aqueous extracts were neutralized with HCl, and the resulting solid was filtered. The cake was dissolved in 3 M HCl and washed with hexanes (40 mL). The aqueous phase was then neutralized with NaOH, and the solid filtered and dried to yield 10.3 g (89%) of **1a** (HYL001) as an off-white solid.

*Route b.* From **5b**: 6-Bromo-4-(2-(6-methylpyridin-2-yl)-5,6-dihydro-4*H*-pyrrolo[1,2-*b*]pyrazol-3-yl)quinoline (**5b**, 9.23 g, 22.8 mmol), Pd_2_(dba)_3_ (522 mg, 0.57 mmol), KOH (3.0 g, 45.6 mmol), and di*tert*-butyl(2′,4′,6′-triisopropyl-3,4,5,6-tetramethyl-[1,1′-biphenyl]-2 yl)phosphine (tetramethyl di-tBuXPhos) (548 mg, 1.14 mmol) were introduced in a flame-dried Schlenk flask and dissolved in degassed dioxane (19 mL) and degassed water (9.5 mL). The mixture was vigorously stirred at 100 °C for 4.5 h; then it was cooled to rt, and 40 mL of a 20% NaOH solution were added. After 30 min of stirring, the suspension was filtered, the solid was washed with NaOH (20%, 20 mL), and the resulting cake was dissolved in HCl (37%, 5 mL). To this solution, 2 g of activated charcoal were added, and the resulting black suspension was stirred for 1 h at reflux, cooled down to rt, filtered, and washed with water. The aqueous solution was neutralized to pH 7 and filtered. The resulting solid was again dissolved in hot MeOH (450 mL), activated charcoal was added, and the suspension was refluxed for 1 h. The mixture was filtered hot, and the filtrate was distilled to the minimum volume and cooled to 0 °C for 1 h. The off-white solid was filtered, washed with cold MeOH, and dried under a vacuum to yield 5.45 g (70%) of **1a** (HYL001) as a white solid. The spectroscopic properties were identical to the ones described;^48^^1^H NMR (400 MHz, DMSO) δ 9.66 (s, 1H), 8.58 (d, *J* = 4.4 Hz, 1H), 7.86 (d, *J* = 9.0 Hz, 1H), 7.56 (t, *J* = 7.7 Hz, 1H), 7.48 (d, *J* = 7.8 Hz, 1H), 7.29–7.16 (m, 2H), 6.96 (d, *J* = 7.5 Hz, 1H), 6.91 (d, *J* = 2.7 Hz, 1H), 4.27 (t, *J* = 7.2 Hz, 2H), 2.79 (t, *J* = 6.5 Hz, 2H), 2.69–2.55 (m, 2H), 1.89 (s, 3H) ppm. ^13^C NMR (101 MHz, DMSO) δ: 156.5, 155.1, 152.0, 151.7, 146.6, 146.2, 143.2, 139.4, 136.5, 130.6, 128.7, 122.5, 121.2, 117.7, 109.9, 107.0, 47.9, 25.5, 23.4, 22.5 ppm. IR (film): 3412, 2949, 2833, 1650, 1618, 1508, 1236, 1010 cm^–1^. HRMS (ESI): *m*/*z* [M + H]^+^ calcd for C_21_H_18_N_4_O, 342.1481; found, 342.1480. The initial attempt (Miyaura borylation) from **5b** is described in the Supporting Methods.

### *In Vitro* Cellular TGFβ Reporter Activity Assays

HEK293T cells were purchased from the ATCC and cultured in DMEM supplemented with l-glutamine and 10% fetal bovine serum (Life Technologies) at 37 °C and 5% CO_2._ The cells were seeded in 24-well plates and transfected with plasmids encoding 12xCAGA-Firefly_Luc and Tk-Renilla_Luc (75 and 10 ng per well, respectively) using polyethylenimine (Polysciences) as the transfection reagent. After 7 h, the medium was replaced with starvation medium (DMEM + 0.05% FBS). The next day, cells were treated with galunisertib, HYL001, or vactosertib, from a 10 mM stock solution in DMSO, in half-log dilutions (0.1–10,000 nM), as well as 5 ng/mL recombinant human TGFB1 (Peprotech). Luciferase activity was measured 16 h later using the Dual Luciferase Assay kit (Promega): media were aspirated, and cells were lysed in 200 μL of passive lysis buffer (kit) for 20 min. Bioluminescence was measured in a Berthold Lumat LB6507 luminometer (18 μL of reagents, 10 s measurements). Results were normalized to those of the Renilla transfection control. Inhibition–concentration curves were analyzed in Prism Graphpad Prism (v10.1.1) with a least-squares regression nonlinear fit.

Further *in vitro* characterizations through CRO’s are described in the Supporting Methods. These include kinase affinity/selectivity and activity, PPB, metabolic stability tests in primary hepatocytes, safety pharmacology, and mutagenic potential.

### Animal Experiments

All procedures involving animal experimentation conducted at Sai Life Sciences Lt. were carried out in accordance with the guidelines provided by the Committee for the Purpose of Control and Supervision of Experiments on Animals (CPCSEA) and after approval by the Institutional Animal Ethics Committee. The study procedures and husbandry care of the study animals were performed in compliance with the Association for Assessment and Accreditation of Laboratory Animal Care (AAALAC) (Unit no. 001384) and CPCSEA (Reg. no. 2121/PO/Rc/S/21/CPCSEA) norms. *In vivo* characterizations (pharmacokinetics and toxicology) through CRO’s are described in the Supporting Methods.

Animal experiments conducted at the IRB Barcelona were approved by the Animal Care and Use Committee of Barcelona Science Park and the Catalan Government (protocol 9162). Mice were maintained in a specific-pathogen-free (SPF) facility with a 12 h light–dark cycle, under controlled temperature and humidity (18–23 °C and 40–60%, respectively) and given ad libitum access to standard diet and water. All mice were closely monitored by authors, facility technicians (during treatments), and an external veterinary scientist responsible for animal welfare.

### Mouse Injections

For all tumor cell injections, C57BL/6J (or athymic BALB/C *nu/nu*) mice were purchased from Janvier at 6 weeks of age and injected at 7–8 weeks. Sex was matched with the origin of the tumor organoids, *i.e.,* males for this study. Intrasplenic or portal vein injections, respectively, with 31G needles on 1 mL insulin syringes or 33G needles on a 50/100 μL Hamilton syringe, were used for liver colonization by the introduction of dissociated organoids (single cells) into the portal circulation. MTOs were cultured in standard six-well plates for 4 days and trypsinized as described before.^5^ The resulting single cell suspension in HBSS (Lonza) was filtered through 100 and 40 μm mesh (to remove clumps of cells and aggregated debris). Intrasplenic injections were performed, as previously described,^5,8,9^ using 2 × 10^5^ cells in 70 μL HBSS. For portal vein injections, 2.5 × 10^3^ cells in 25 μL HBSS were injected directly into the portal vein.^5^ Mice were euthanized at 3–5 weeks or at the humane end point (advanced metastasis-associated morbidity causing or threatening to cause severe suffering) to obtain survival-type data. Visible liver metastases were counted, and data were analyzed using GraphPad Prism software (v.7.03).

### Mouse Treatment

HYL001, galunisertib, vactosertib, or control vehicle were administered by gavage twice per day, starting at the indicated time points after cell injection. Gavage treatments were performed by technicians from the animal facility, not involved in this study (blinded). For checkpoint immunotherapy or dual treatments, we used rat anti-PD-1 (RMP1–14; Leinco P372) or rat IgG2a (Leinco I-1177) isotype-control antibodies.

Experimental group sizes were practically associated with cage sizes (5 mice/cage) and experiments were designed to have *n* ≥ 5 per group (1 or more cages) and repeated at least once in independent experiments, as in.^5^ No mice were excluded from the analysis. For gavage treatment, as the control vehicle and compound containing vials were visually distinguishable, the only randomization we performed was the order of injecting mice: the researcher performing the injections was blinded to the treatment group. End point criteria are equivalent to those described above.

## Supporting Information Available

Alternative synthesis of HYL001 (**1a**) from **5b**, synthesis of HYL002 and HYL002-HCL3, ^1^H and ^13^C NMR of all new compounds, HPLC of HYL001 (**1a**), galunisertib (**1b**), and vactosertib (**2**), methods for *in vitro* characterization of HYL001, methods for *in vivo* characterizations of HYL001, irinotecan, and SN-38, synthesis of pro-drug HYL002 from HYL001, plasma concentrations of HYL001 in rodents, i*n vitro* safety pharmacology of HYL001, i*n vivo* reduction of TGFβ target protein levels by HYL001, PPB of HYL001 and warfarin, metabolic stability of HYL001 in the presence of human hepatocytes, hERG inhibition, cardiac toxicity assessment for HYL001, mutagenic potential of HYL001, and selectivity of HYL001, galunisertib, and vactosertib to ALK family members (PDF)
