# Supplementary material for: New Potent Inhibitor of Transforming Growth Factor-Beta (TGFβ) Signaling that is Efficacious against Microsatellite Stable Colorectal Cancer Metastasis in Combination with Immune Checkpoint Therapy in Mice
Source: ACS Pharmacol Transl Sci. 2024 Oct 1;8(1):97–112. doi: 10.1021/acsptsci.4c00374 (PMC11729428; doi:10.1021/acsptsci.4c00374)
Supplement: Supplementary file 1 — pt4c00374_si_001.pdf [file pt4c00374_si_001.pdf]

Supporting Information

New potent inhibidor of Transforming Growth Factor-beta (TGFβ) signalling that is efficacious against microsatellite stable colorectal cancer metastasis in combination with immune checkpoint therapy in mice

Daniele V. F. Tauriello[1,2,3§], Elena Sancho[1,2§], Daniel Byrom[1§], Carolina Sanchez-Zarzalejo[1], Maria Salvany[1,2], Ana Henriques[1], Sergio Palomo-Ponce[1,2], Marta Sevillano[1,2], Xavier Hernando-Momblona[1,2], Joan A. Matarin[1], Israel Ramos[1], Irene Ruano[1], Neus Prats[1], Eduard Batlle[1,2,5*], Antoni Riera[1,4*]*

d.tauriello@erasmusmc.nl; eduard.batlle@irbbarcelona.org; ariera@ub.edu

1. Institute for Research in Biomedicine (IRB Barcelona), The Barcelona Institute of Science and Technology (BIST), Baldiri i Reixac 10, 08028 Barcelona, Spain
2. Centro de Investigación Biomédica en Red de Cáncer (CIBERONC), 08028 Barcelona, Spain.
3. Department of Medical Oncology, Erasmus MC Cancer Institute, University Medical Center Rotterdam, 3015 GD Rotterdam, The Netherlands
4. Department Química Inorgànica i Orgànica, Universitat de Barcelona, Martí i Franquès 1, 08028 Barcelona, Spain
5. Institució Catalana de Recerca i Estudis Avançats (ICREA), 08010 Barcelona, Spain.

[§] *Contributed equally to this work* * *Corresponding Authors*

1. Alternative synthesis of HYL001 (**1a**) from **5b** S-2

2. Synthesis of HYL002 and HYL002·HCl S-3

3. ^{1}H and ^{13}C NMR of HYL001 (**1a**) and HYL002 ....S-5

4. HPLC of HYL001 (**1a**), galunisertib (**1b**) and vactosertib (**2**) S-9

5. Methods for *in vitro* characterization of HYL001 ....S-12

6. Methods for *in vivo* characterization of HYL001 ....S-16

7. Supplementary Figures S1–S4 and Schemes S1 ....S-19

8. Supplementary Tables S1–S5 ....S-22

1.- Alternative synthesis of HYL001 (1a) from 5b.

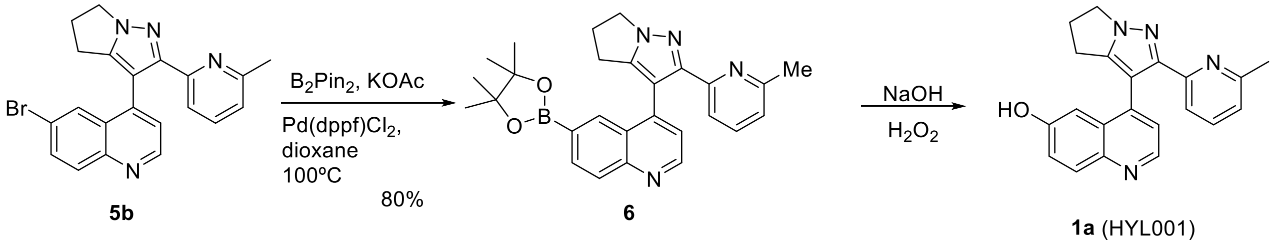

(Miyaura borylation): To a flame-dried flask, 6-bromo-4-(2-(6-methylpyridin-2-yl)-5,6-dihydro-4H-pyrrolo[1,2-b]pyrazol-3-yl)quinoline (**5b**, purified by chromatography, 7.15 g, 17.6 mmol), Pd(dppf)Cl_2 (644 mg, 0.88 mmol), KOAc (5.19 g, 52.9 mmol) and bis(pinacolato)diboron (5.38 g, 21.2 mmol) were added under N_2. Degassed dioxane (140 mL) was added and the mixture heated to 90 ºC for 3 h. The reaction was slowly cooled to 0 ºC and diluted with NaOH (5M, 35.3 mL, 176 mmol). Then, a mixture of 30% H_2O_2 (18 mL, 176 mmol) and water (36 mL) was added dropwise. After 18 h at rt, the reaction was cooled to 0 ºC and quenched with sodium sulphite (2 M, 83 mL, 176 mmol). After stirring for 30 minutes, the reaction mixture was checked for the presence of peroxides, filtered and concentrated under vacuum. The resulting aqueous solution was neutralized with HCl and cooled down to 0 ºC. The solid was filtered, washed with water and dissolved again in NaOH 5M (70 mL). This solution was refluxed with 2 g of activated carbon for 1 h, filtered and neutralized

with HCl. The resulting solid was filtered, washed with water and dissolved in HCl 1M (35 mL), then treated with a 20% solution of N-acetylcysteine for 5 h. The solid was then precipitated with acid, filtered, dissolved in hot MeOH (300 mL) and refluxed with 1 g of activated charcoal for 1 h. The solution was filtered hot and the solvent was removed by distillation to the minimum volume. The resulting white suspension was cooled at 0 ºC for 2 h and filtered to yield 4.81 g (80%) of **1a** (HYL001) as a white solid.

2.-Synthesis of HYL002.

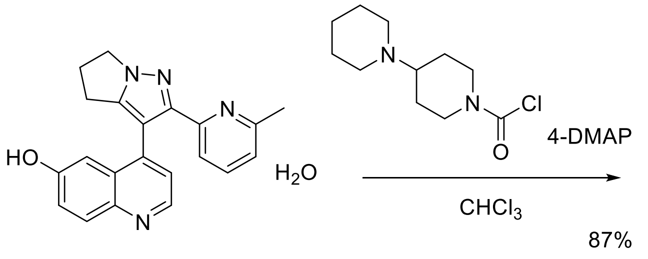

A solution of HYL001 (47 mg, 0.14 mmol) in 3 mL of $CHCl_3$ was prepared and transferred under N_2 to a Schlenk flask containing 3 Å activated molecular sieves. The solution was stirred at room temperature for 1 h. A solution of [1,4'-bipiperidine]-1'-carbonyl chloride (32 mg, 0.14 mmol, 1 eq.), Et_3N (23 µL, 0.17 mmol, 1.2 eq.) and a crystal of 4-dimethylaminopyridine in 1 mL of $CHCl_3$ was then added dropwise. The resulting mixture was stirred overnight. Some starting material was detected by TLC the following day, so another solution of [1,4'-bipiperidine]-1'-carbonyl chloride (16 mg, 0.07 mmol, 0.5 eq.), Et_3N (12 µL, 0.08 mmol, 0.6 eq.) and a crystal of 4-dimethylaminopyridine in 0.5 mL of $CHCl_3$ was added to the reaction mixture and it was stirred overnight. The reaction was quenched by adding 20 mL of H_2O. The resulting aqueous layer was extracted with EtOAc (3 x 20 mL), dried over $MgSO_4$ and concentrated to afford the crude product which was purified by column chromatography (eluted with a gradient of 10-20% MeOH in DCM) to obtain 64 mg (87%) of the **(2**-HYL002 ((6-methylpyridin-2-yl)-5,6-dihydro-4H-pyrrolo[1,2-b]pyrazol-3-yl)quinolin-6-yl [1,4'-bipiperidine]-1'-carboxylate) as an orange oil.

^{1}H NMR (400 MHz, $CDCl_3$) δ 8.79 (dd, *J* = 4.5, 1.4 Hz, 1H), 8.09 (dd, *J* = 9.1, 1.2 Hz, 1H), 7.49 – 7.39 (m, 2H), 7.31 – 7.23 (m, 2H), 6.92 (dd, *J* = 11.4, 7.7 Hz, 2H), 4.35 – 4.26 (m, 4H), 2.98 – 2.76 (m, 4H), 2.72 – 2.50 (m, 7H), 2.34 (s, 3H), 1.97 – 1.87 (m, 2H), 1.75 – 1.42 (m, 8H) ppm.

^{13}C NMR (101 MHz, $CDCl_3$) δ 158.5, 153.6, 153.3, 151.4, 149.5, 149.3, 147.0, 146.5, 141.2, 136.3, 131.0, 128.1, 125.0, 123.0, 121.9, 119.2, 117.2, 110.0, 62.7, 50.3, 48.5, 26.1, 25.8, 24.5, 24.4, 23.2 ppm.

Synthesis of HYL002·HCl

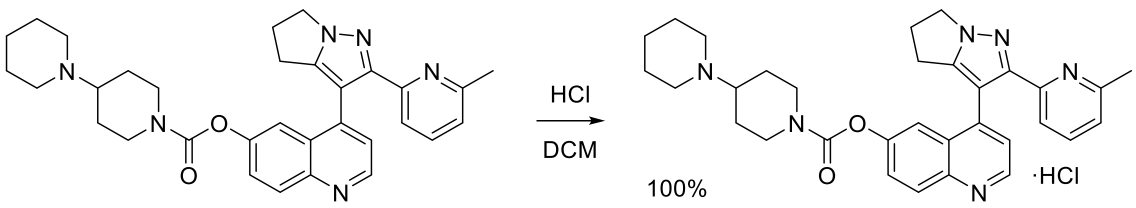

HYL002 (65 mg, 0.12 mmol) was dissolved in DCM (1.5 mL) and treated with 0.3 mL (1.21 mmol) of a 2 M solution of HCl in dioxane during 2 h. Then the solvent was removed to afford HYL002·HCl as a pale orange solid (70 mg, 100%).

^{1}H NMR (400 MHz, Methanol-d_4) δ 9.18 (d, *J* = 5.6 Hz, 1H), 8.35 (d, *J* = 9.2 Hz, 1H), 8.12 (d, *J* = 5.6 Hz, 1H), 8.06 (t, *J* = 7.9 Hz, 1H), 8.02 – 7.93 (m, 2H), 7.74 (d, *J* = 7.9 Hz, 1H), 7.28 (d, *J* = 7.9 Hz, 1H), 4.56 – 4.39 (m, 3H), 4.31 – 4.28 (m, 1H), 3.59 – 3.56 (m, 2H), 3.52 – 3.45 (m, 2H), 3.19 – 2.95 (m, 6H), 2.88 (s, 3H), 2.85 – 2.81 (m, 1H), 2.25 – 2.20 (m, 2H), 2.01 – 1.85 (m, 7H), 1.60 – 1.50 (m, 1H) ppm.

^{13}C NMR (101 MHz, Methanol-d_4) δ 157.0, 153.3, 153.2, 152.2, 151.0, 147.2, 146.1, 145.5, 144.9, 137.62, 131.4, 129.3, 128.0, 124.6, 124.5, 124.1, 119.5, 111.0, 66.9, 64.6, 51.4, 50.4, 44.3, 44.0, 27.5, 27.2, 27.1, 24.5, 24.2, 22.9, 20.1, 15.5 ppm.

3. ^{1}H and ^{13}C NMR of **HYL001** (**1a**) and **HYL002**

^{1}H NMR HYL001

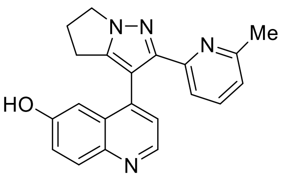

HYL001

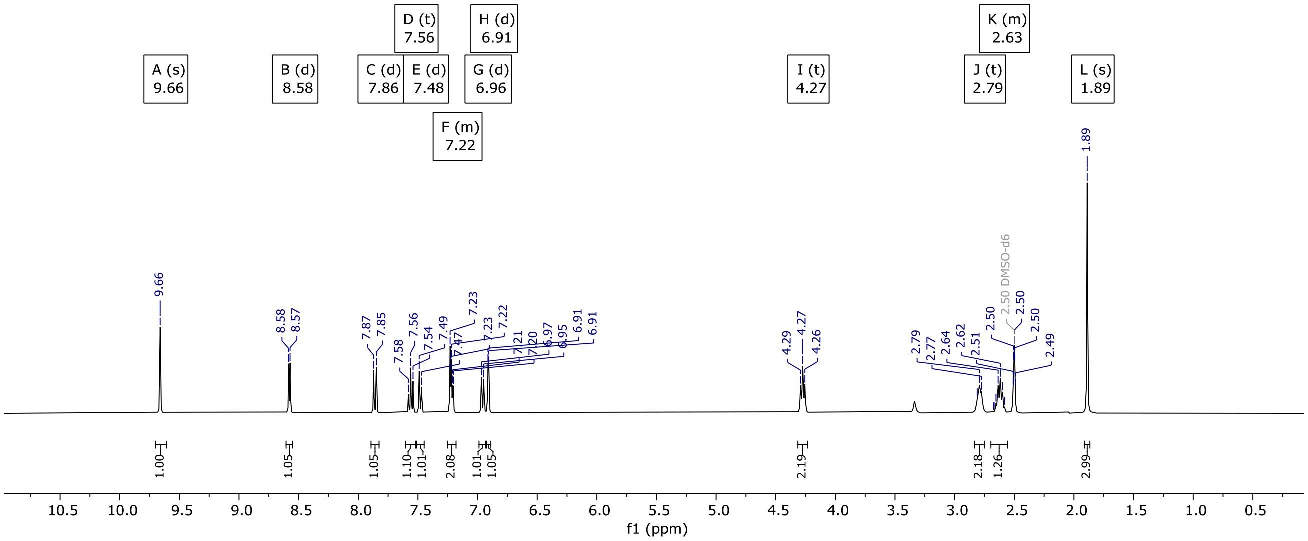

^{13}C NMR HYL001

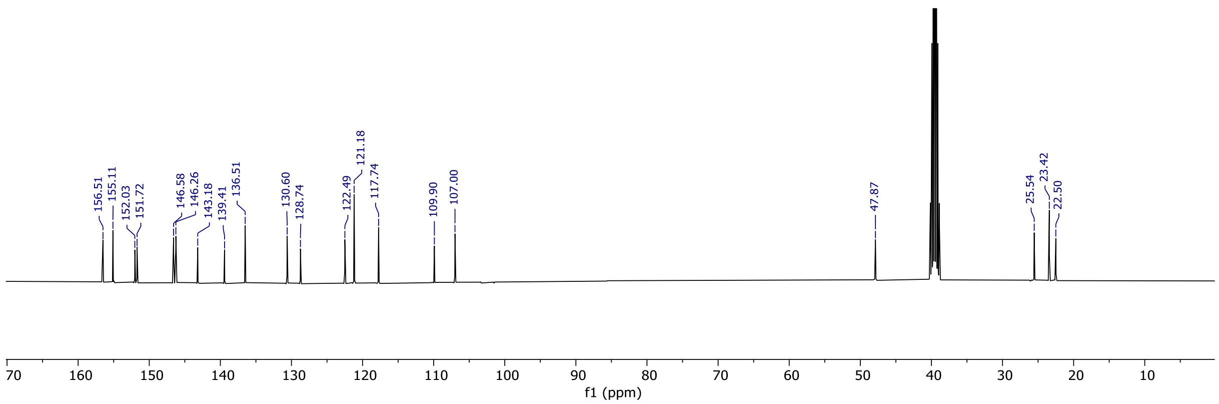

^{1}H NMR HYL002

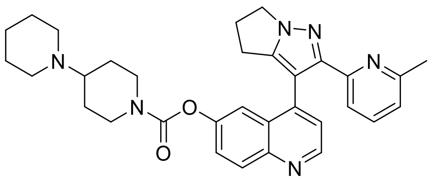

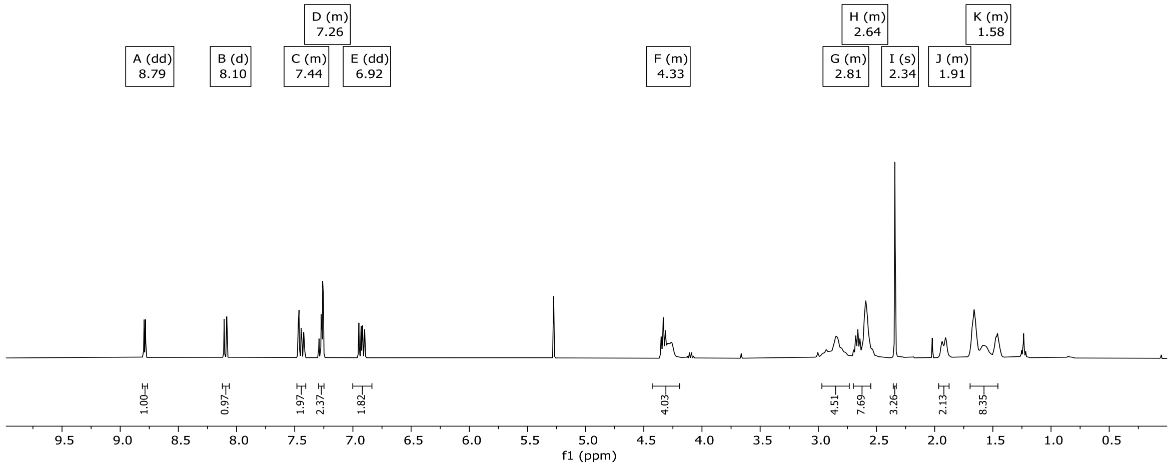

^{13}C NMR HYL002

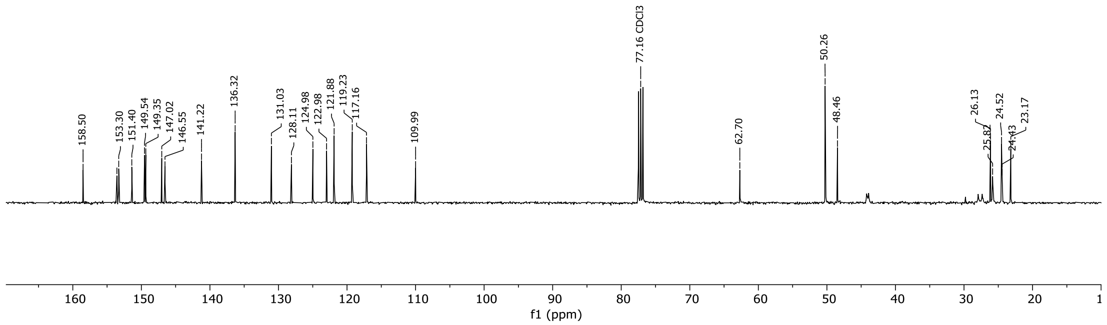

^{1}H NMR HYL002·HCl

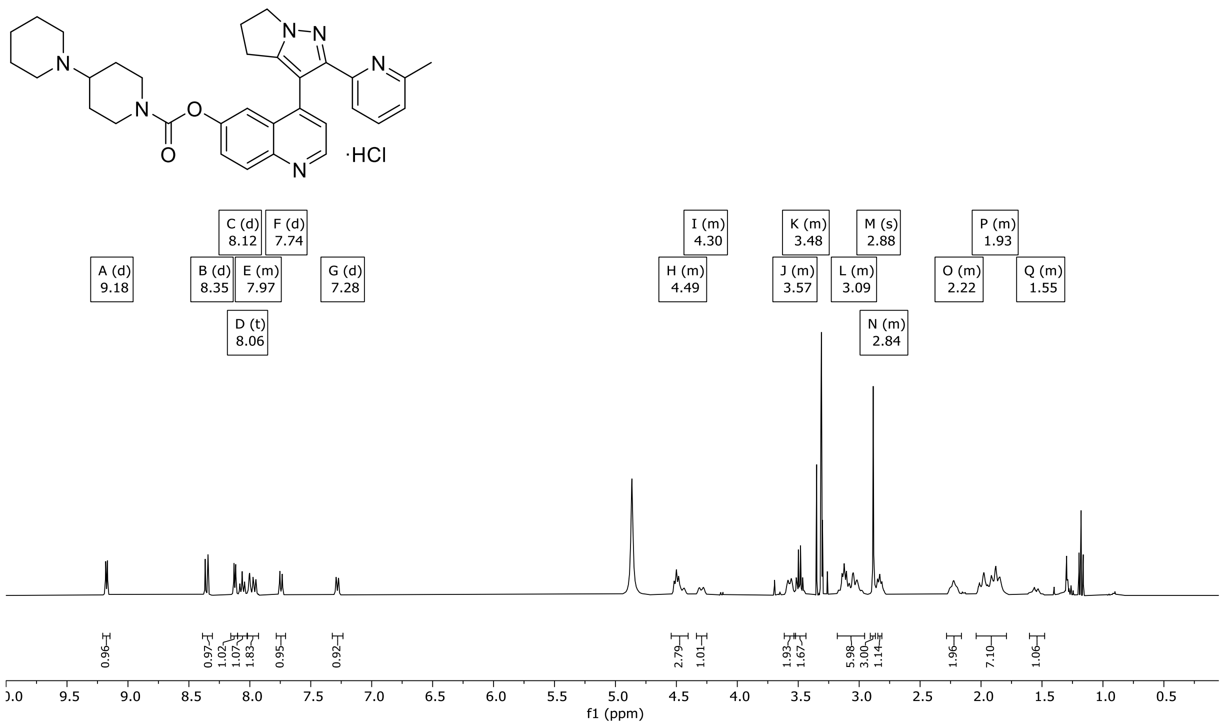

^{13}C NMR HYL002·HCl

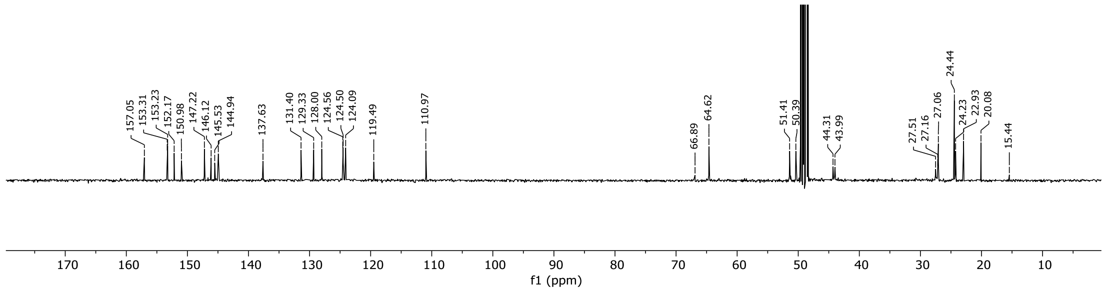

4. HPLC of HYL001 (1a), galunisertib (1b) and vactosertib (2)

HPLC of HYL001

Kinetex EVO C18 50x 4.6mm, 2.6um; Mobile phase: 10mM NH4HCO3 pH8 / ACN (
95:5)--0.5min---(95:5)---6.5min----(0:100)---2min----(0:100) post run 1.5
min; Tª40°C

Aprox. 1 mg/ml MeOH
Walkup method: 'M1_Positive_Standard_9min'
Target:

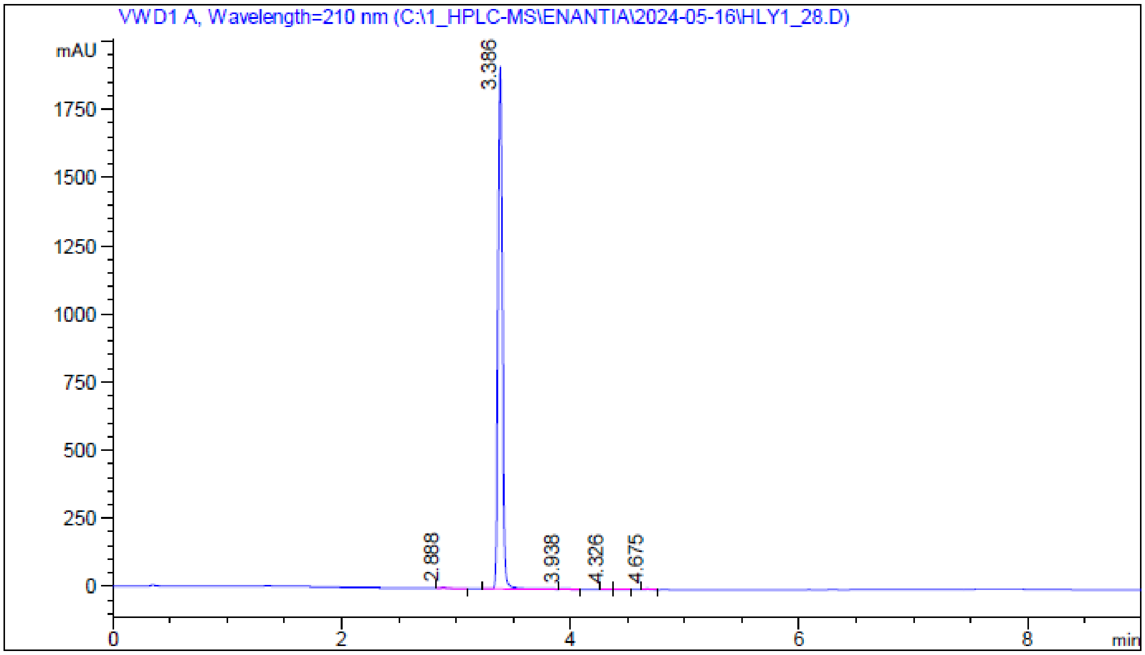

Signal: 1-> : VWD1 A, Wavelength=210 nm

#	Meas. Ret. Time	Area	Area %
1	2.888	13.72	0.26
2	3.386	5178.13	99.30
3	3.938	7.18	0.14
4	4.326	4.54	0.09
5	4.410	4.55	0.09
6	4.675	6.65	0.13

HPLC of galunisertib (1b**)**

```
Info mostra:    HPLC8, Column XBridge C18 150 x 4.6 mm, 3.5 um (COL-HP-138)
                ;
                H2O :ACN (90:10)---20min---(0:100) post time 5min
                1mg/mL MeOH
```

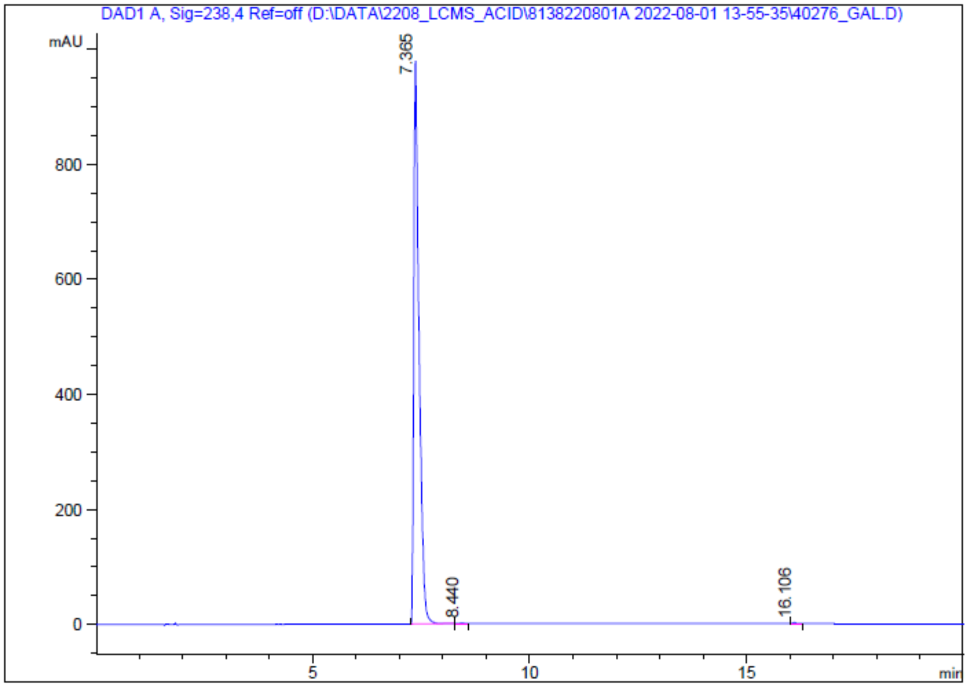

#	Meas. R	Pea	Width	Area	Height	Area %
1	7.365	BB	0.116	8217.666	978.557	99.825
2	8.440	BB	0.088	6.648	1.213	0.081
3	16.106	BB	0.067	7.722	1.777	0.094

HPLC of vactosertib (2)

Kinetex EVO C18 50x 4.6mm, 2.6um; Mobile phase: 10mM NH4HCO3 pH8 / ACN (95:5)--0.5min---(95:5)---6.5min----(0:100)---2min----(0:100) post run 1.5 min; Tª40°C

Aprox. 1 mg/ml MeOH
Walkup method: 'M1_Positive_Standard_9min'
Target:

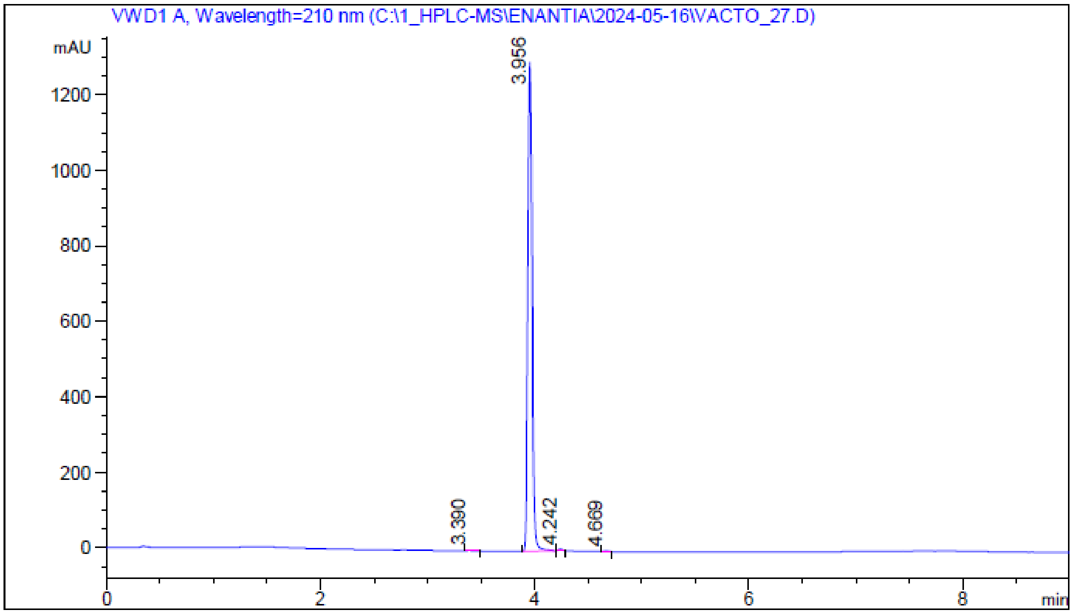

Signal: 1-> : VWD1 A, Wavelength=210 nm

#	Meas. Ret. Time	Area	Area %
1	3.390	3.57	0.10
2	3.956	3545.47	99.34
3	4.242	11.93	0.33
4	4.669	7.96	0.22

5. Methods for *In vitro* characterizations of HYL001

Kinase affinity and selectivity

The affinity of the ALK5 inhibitor HYL001 was determined for the ALK kinase family using the KINOMEscan™ Technology—measuring the ability of compounds to competitively inhibit binding between DNA-tagged kinases and immobilized assay ligands, using quantitative PCR—run by Eurofins/DiscoveryX (Birmingham AL, USA). Dissociation constants were calculated by measuring the amount of kinase captured on the solid support as a function of the test compound concentration. K_d values were obtained by an 11-point half-log dilution range of 0.5–30,000 nM of inhibitor for ALK1 (ACVRL1), ALK2 (ACVR1), ALK3 (BMPR1A), ALK4 (ACVR1B), ALK6 (BMPR1B), ACVR2B and TGFβR2. In addition, this technology was used to assess selectivity towards a panel of 97 Kinases (scanEDGE) by Eurofins Panlabs (Grapevine TX, USA). The strength of competitive binding, defined as the percentage of binding inhibition of kinases to assay ligand, was visualized using the TREEspot software (DiscoverRx Corporation) and a selectivity score was calculated (the fraction of non-mutated kinases tested with competitive binding inhibition above threshold percentages 65 or 90).

Enzymatic activity

The activity, measured as IC_{50}, of inhibitors was determined by radiometric γ-33P-ATP-mediated *in vitro* phosphorylation assays, using the Kinase Profiler service from Eurofins Pharma Discovery Services (Wolverhampton, UK) and Eurofins Cerep (Celle L'Evescault, France). Compounds, prepared in 100% DMSO, were tested using a 9-point curve with half-log serial dilutions (1–10,000 nM). For recombinant TGFBR1, the fragment 200–end (T204D) was used; p38alpha was used as a full-length recombinant protein.

Plasma protein binding

Rapid equilibrium dialysis was performed in triplicates with a rapid equilibrium dialysis (RED) by Sai Life Sciences Lt. (Telangana, India). The RED device contains a dialysis membrane with a molecular weight cut-off

of 8,000 Da, separating two chambers for plasma and buffer, respectively. 200 uL of 5 µM warfarin or HYL001 were added to the plasma chamber, versus phosphate buffer saline (pH 7.4) to the buffer chamber. Dialysis was performed for 4 h with 100 RPM shaking at 37°C. To assess recovery and stability, aliquots of warfarin or test compounds in plasma were either frozen immediately (T0 sample), or incubated at 37°C for 4 h without dialysis (non-dialysed), respectively.

Following dialysis, an aliquot was removed from either chamber and diluted with equal volume of opposite matrix to nullify the matrix effect. Similarly, buffer was added to recovery and stability samples. Next, acetonitrile (containing internal standard, glipizide) was added to the mixtures for protein precipitation and vortexed for 5 minutes. The samples were centrifuged at 4000 RPM at 4°C for 10 min and the supernatant was submitted for liquid chromatography mass spectrometry (LC-MS/MS) analysis. Samples were monitored for parent compound using multiple reaction monitoring (MRM) mode.

The peak area ratios (PAR, analyte versus internal standard) in plasma *vs* bufffer were used to determine the fraction of compound bound to plasma proteins. The following equation was used to determine the extent of plasma protein binding: percent free drug = 100% x $(PAR_{buffer} / PAR_{plasma})$; percent bound drug = 100 - % free drug. Recovery was calculated as 100% x $(PAR_{plasma} + PAR_{buffer})_{dialysed} / (PAR_{plasma})_{non\text{-}dialysed}$; stability as 100% x $(PAR)_{non\text{-}dialysed} / (PAR_{plasma})_{frozen\ at\ T0}$.

Metabolic stability in human primary hepatocytes

This assay was performed at Sai Life Sciences Lt. (Telangana, India) with human suspension hepatocytes (HMCS1S, Gibco USA). 200 µL of cell suspension containing 2 x 10^{6} hepatocytes/mL (>95% viability) was added to individual wells of 24 well plates and incubated in a CO_2 incubator at 37°C and 5% CO_2 for 15 min. T0 incubation was terminated by adding 1000 µL of ice-cold acetonitrile. All other reactions were initiated by adding 200 µL of test compound diluted in Krebs Henseleit Buffer (pH 7.4; pre-warmed at 37°C in CO_2 incubator) and further incubated for 0, 15, and 60 min before being terminated as the T0 sample. Samples were sonicated for 5 min before centrifuging at 2147 g for 15 min at 4°C, and the supernatant was submitted for

analysis by LC-MS/MS, using MRM mode. Testosterone and 7-OH coumarin were used as positive controls related to phase I and phase II metabolism, respectively.

The PARs were used for calculation of metabolic stability. The 0 min area ratio was considered as 100%. The slope of the initial linear range of logarithmic curve of percent remaining versus time (elimination constant K_e) was used for calculation of half-life: $T_{1/2} = \ln 2 / K_e$, as well as intrinsic clearance (μL/min/million cells): incubation volume / number of cells (in millions) x K_e. To calculate into full body context, the values of 120 million cells/g liver and 20 g liver/kg body weight were used.

In vitro *safety pharmacology*

HYL001 was tested at 10 μM for inhibition of binding and in enzymatic activity of relevant proteins linked to adverse drug reaction risks, using the SafetyScreen 44 service from Eurofins (Celle L'Evescault, France). In each experiment, the respective reference compound was tested concurrently with HYL001, and the data were compared with historical values determined at Eurofins. Binding was defined as the % inhibition of the signal of a radioactively labelled ligand specific for each target, and enzymatic activity as the % inhibition of control enzyme activity. Values are shown as the mean of technical duplicates, error bars represent SD. Results showing an inhibition or stimulation higher than 50% are considered to represent significant effects of the test compound.

The potential degree of hERG inhibition by HYL001 at 3 concentrations was measured by automated patch clamp (Eurofins, Celle L'Evescault, France) on CHO-K1 cells. The tail current amplitude, induced by a one second test pulse to -40 mV after a two second pulse to +20 mV, was measured before and after drug incubation and expressed as % inhibition.

Mutagenic potential

The Ames test was performed at Xenomatrix (Allschwil, Switzerland). Bacteria from the *S. typhimurium* strains TA98, TA100, TA1535 and TA1537 were exposed to 6 concentrations of a HYL001 (1–320 μg/ml; 3–934 μM; performed in triplicates) in the absence or presence of liver S9 extracts, as well as a positive and a

negative control, for 90 minutes in medium containing sufficient histidine to support approximately two cell divisions. After exposure, the cultures were diluted in pH indicator medium lacking histidine or tryptophan and aliquoted into 48 wells of a 384-well plate. Within two days, cells that have undergone reversion to amino acid prototrophy grow into colonies. The number of wells containing revertant colonies were counted by pH indicator colour change for each dose and compared to a solvent (negative) control. A dose dependent increase in the number of revertant colonies upon exposure to HYL001 would have indicated mutagenicity.

6. Methods for *in vivo* characterizations of HYL001

Pharmacokinetics of HYL001 in BALB/c Mice and Sprague Dawley Rats.

The plasma pharmacokinetics of HYL001 in male BALB/c mice and male Sprague Dawley rats were investigated following a single oral administration at 71 mg/kg dose by Sai Life Sciences Lt. (Telangana, India). A total of nine mice were used in this study with 3 mice/time point, following a sparse sampling design. Similarly, three rats were used in this study. Animals were administered orally with a suspension formulation of HYL001 at 71 mg/kg dose. The formulation vehicle was 1% *v/v* NaCMC, 0.4% *v/v* SLS and 0.085% *v/v* PVP 40T in RO water.

Blood samples (approximately 60 μL for mice and approximately 120 μL for rats) were collected under light isoflurane anesthesia (Surgivet) from retro orbital plexus from a set of three mice at Pre-dose, 0.25, 0.5, 1, 2, 4, 6, 8, and 24 hr. Immediately after blood collection, plasma was harvested by centrifugation at 4000 rpm, 10 min at 4^0C and samples were stored at -70 ±10ºC until bioanalysis.

All samples were processed for analysis by protein precipitation method and analyzed by LC-MS/MS. The plasma pharmacokinetic parameters were estimated using non-compartmental analysis tool of Phoenix® WinNonlin software (Version 8.0)

Toxicology Studies in Rats and Mice

Sprague-Dawley rats were procured from Hylasco Bio-Technology Pvt. Ltd., Hyderabad. After completion of the acclimatization period twelve healthy rats were randomly allocated to the control and treatment groups. Treatment groups included controls, and either HYL001 (25 mg/kg BID or 77 mg/kg BID) or Vactosertib (82 mg/kg BID) treated animals, having three rats/sex/group. Rats from control group received vehicle, consisting of 0.4 % Sodium Lauryl Sulphate (SLS), 0.085 % Povidone (Polyvinylpyrrolidone), 0.05% Antifoam A and quantity sufficient to 1% w/v Carboxy Methyl Cellulose Sodium Salt. Rats from treatment groups were administered with suspensions of the indicated drugs in vehicle, orally twice a day for an intended period of 28

consecutive days. The dosing volume was kept constant at 10 mL/kg for each rat. Rats that showed any critical clinical sign that worsened during the treatment, were necropsied in the shortest time for ethical reasons. The rest of the rats were necropsied at the end of the treatment.

Following 28 days of treatment, or when found sick, the rats were humanely euthanized by carbon dioxide asphyxiation on day 29, except for one group of HYL001 treated rats with 71 mg/kg BID, that were allowed to recover from treatment for further 28 days.

The rats were subjected to detailed gross pathological examination which included careful examination of the external surface of the body, all orifices and the cranial, thoracic and abdominal cavities and their contents. After gross pathological examination the vital organs were trimmed of any adherent tissue and were weighed wet. Paired organs were weighed together. Organ weights relative to terminal body weights were calculated for each rat.

Parameters evaluated during the study included in-life observations such as clinical signs observation, body weights, percent body weight gains, and feed consumption. Parameters like hematology, clinical chemistry, gross pathology, and organ weights were evaluated on the day of termination. Several organs/tissues were further processed and evaluated for histopathology, where special attention was given to bones, gastrointestinal system (stomach, duodenum, jejunum, ileum, caecum, colon and rectum), heart, and vascular lesions in the aorta and coronary arteries. For histopathology, 3-5 μM thick tissue sections were cut and stained with hematoxylin-eosin stain. The heart was trimmed by longitudinally bisecting along a plane perpendicular to the plane of the pulmonary artery to expose the right atrioventricular, left atrioventricular, and aortic valves. Both halves of the heart were embedded as hemisection in paraffin with the cut surface down. Blocks were sectioned to obtain at least three heart valves.

All the individual animal data were summarized in terms of group mean and standard deviation. Body weight, body weight gain, hematology, clinical chemistry, organ weight data of toxicity group rats were analyzed using a T-test. All analysis and comparisons were evaluated at 5% level i.e. $P \leq 0.05$. The statistical analysis was

performed using GraphPad Prism statistical software version 5.02 for Windows, GraphPad Software, San Diego California USA.

The study design in mice was similar as in rats, except 5 mice of each sex were used (n=10 in total), and that there was not a recovery group. All analyses were performed at day 29 after termination of treatment.

7. Supplementary Figures S1–S4 and Schemes S1

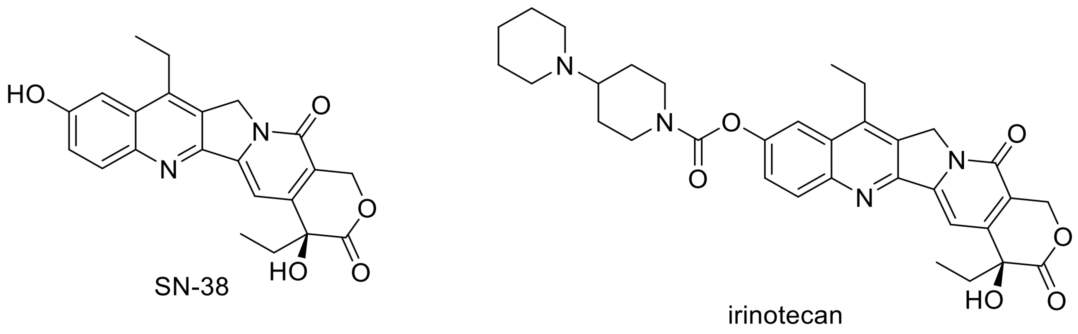

Figure S1. Irinotecan as the carbonyl-4-piperidinopiperidine ester of SN-38.

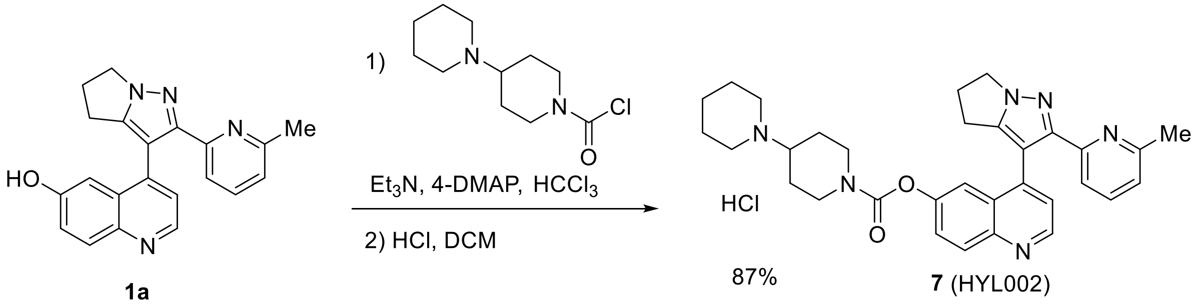

Scheme S1. Synthesis of pro-drug HYL002 from HYL001.

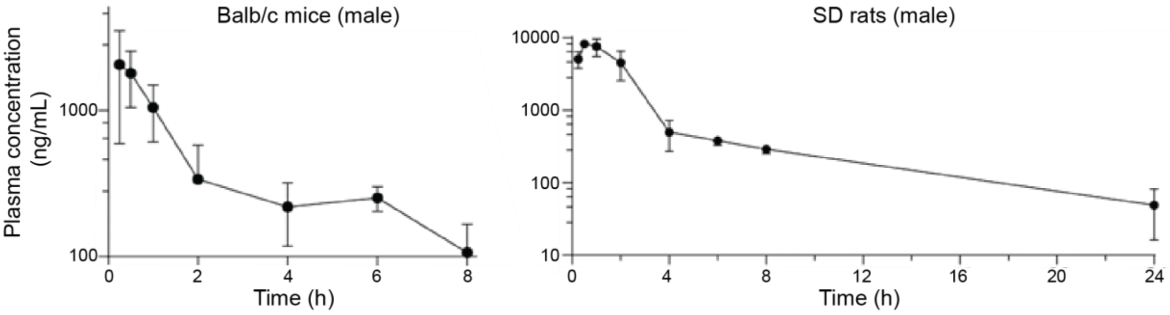

Figure S2. Plasma concentrations of HYL001. Longitudinal measurements after oral administration of a single dose (71 mg/kg) in Balb/c mice and Sprague Dawley (SD) rats. The area under the curve for plasma concentration−time, extrapolated to the last time point after oral dosing (AUC_{last}) was 3520 ng·h/mL for mice and 21575 ng·h/mL for rats

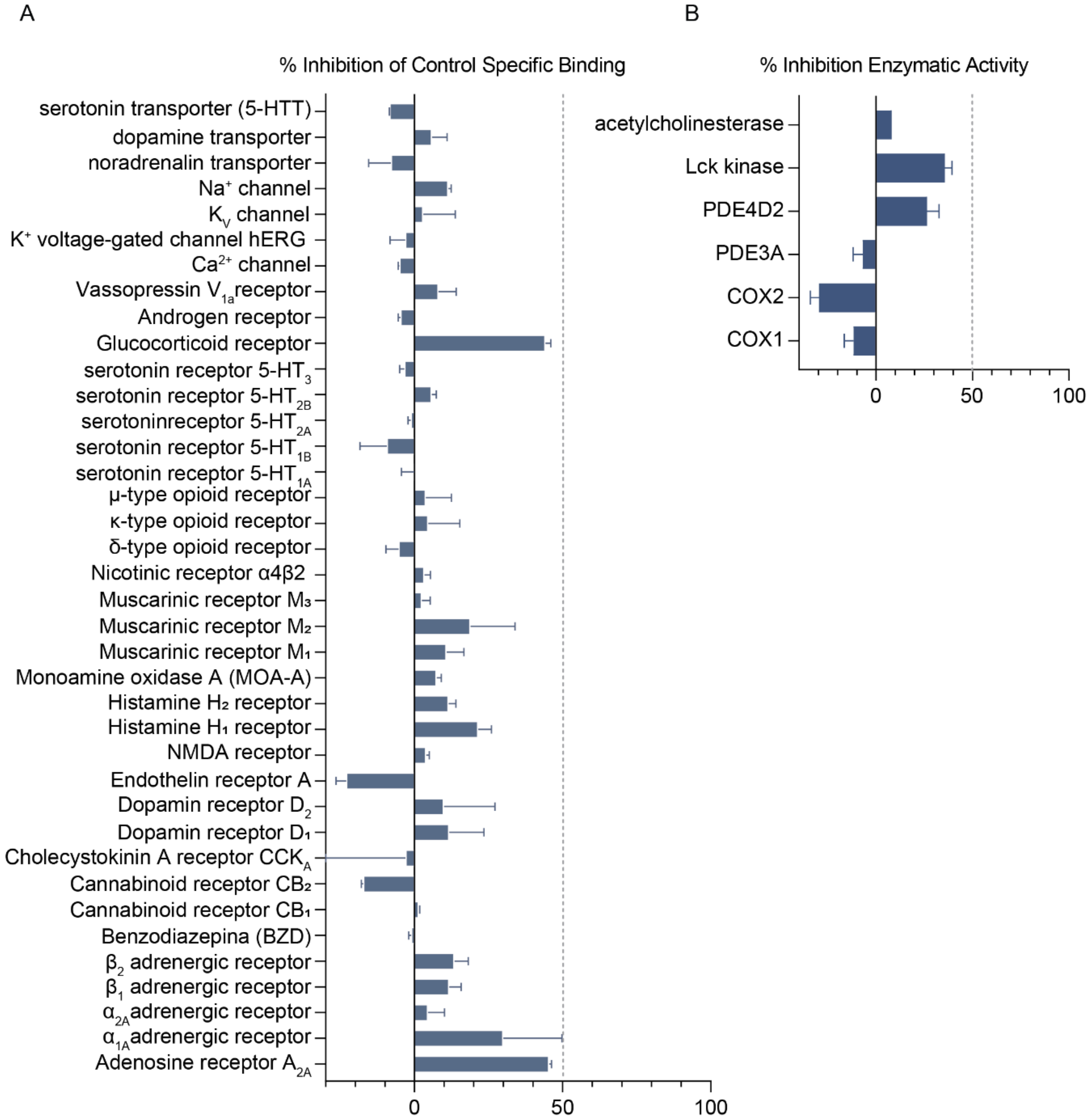

Figure S3. ***In vitro* safety pharmacology**. Binding (**A**) and enzymatic inhibition (**B)** of indicated receptors, protein channels and enzymes by 10 μM HYL001. HYL001 binding was calculated as the % inhibition of the binding of a radioactively labelled ligand specific for each target. HYL001 enzyme inhibition effect was calculated as % inhibition of control enzyme activity. Values are averages of two measurements ±SD.

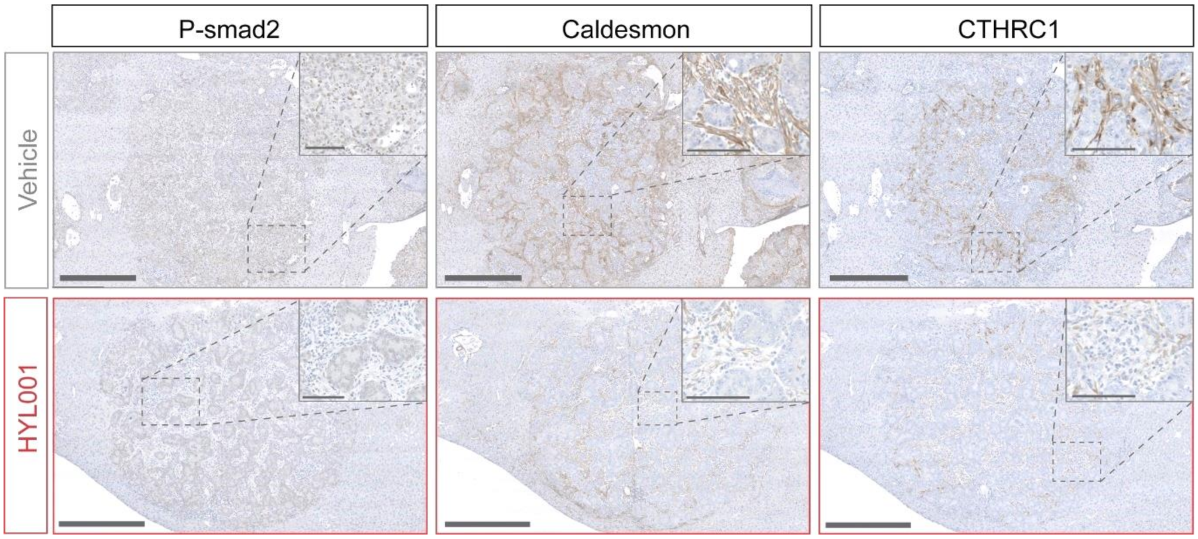

Figure S4. HYL001 treatment reduces protein levels of TGFβ signalling targets *in vivo*. Representative images of the indicated protein expression levels assessed by immunohistochemistry in liver metastases of mice with established liver metastases treated for 3 days with HYL001 at 0.3x dose. Bars 500 μm in 10x magnification images; 100 μm in insets.

8. Supplementary Tables S1–S5

Table S1. Selectivity towards ALK family members.

	Analyses herein			Reported data[15]
	HYL001	Vactosertib	Galunisertib	Galunisertib
ALK1 (ACVRL1)	11000			24900
ALK2 (ACVR1)	7500			35700
ALK3 (BMPR1A)	6900			16800
ALK4 (ACVR1B)	13			77.7
ALK5 (TGFBR1)	20 / 25[a]	4.1	52	172
ALK6 (BMPR1B)	160			471
ACVR2B	100			694
TGFBR2	2000			208

Binding constants (K_d) in nM. [a]This assay was run twice with an average of 22.5 nM.
[Ref. 15](Yingling 2018); see main document

Table S2. Plasma protein binding in 3 species, by equilibrium dialysis (n= 3)

**Compounds**	**Species**	**% Bound** Mean ± SD	**% Free fraction**	**% Recovery**	**% Compound remaining at 4 hrs**
**Warfarin**	Human	99.1 ± 0.1	0.9	100.2	84.2
	Rat	99.1 ± 0.1	0.9	98.6	104.8
	Mice	97.2 ± 0.5	2.8	99.9	106.2
**HYL001**	Human	91.6 ± 1.1	8.4	100.6	101.1
	Rat	95.1 ± 0.8	4.9	100.3	106.1
	Mice	95.2 ± 0.3	4,8	112.4	113.1

Table S3. Metabolic stability assessed with human hepatocytes.

Incubation Time (min)	HYL001 (%)	Galunisertib (%)	Vactosertib (%)
**0**	100	100	100
**5**	67	97	60
**15**	59	92	59
**30**	56	90	52
**60**	38	76	39
**Disappearance $T_{1/2}$ (min)**	51.8	>60*	55.3
**Scaled *in vivo* Cl_{int}(ml/min/kg)**	32.1	10.4	30.1
**Buffer Stability (%)**			
**0**	100	100	100
**60 min**	117	105	84

*Assumed $T_{1/2}$ = 160 min; Cl_{int} predicted with hepatocyte $T_{1/2}$. Scaled Cl_{int} using 120 M cells per g liver (human), liver weight: 20 g/kg (human), human liver blood flow (LBF): 20.7 ml/min/kg.

Table S4. hERG inhibition cardiac toxicity assessment for HYL001.

Concentration (μM)	Inhibition (%)
3.7	1.8
11.1	8.4
33.3	22.8

Table S5. Mutagenic potential assessed in *S. typhimurium* strains.

S. typhimurium strain	Mutagenic data points		Dose dependency	
	w/o S9	with S9	w/o S9	with S9
TA98	No	No	No	No
TA100	No	No	No	No
TA1535	No	No	No	No
TA1537	No	No	No	No
